# Qualitative assessment of facilitators and barriers to HIV programme implementation by community health workers in Mopani district, South Africa

**DOI:** 10.1371/journal.pone.0203081

**Published:** 2018-08-30

**Authors:** Nireshni Naidoo, Nkosinathi Zuma, N. Sellina Khosa, Gert Marincowitz, Jean Railton, Nthabiseng Matlakala, Geoffrey A. Jobson, Jude O. Igumbor, James A. McIntyre, Helen E. Struthers, Remco P. H. Peters

**Affiliations:** 1 Anova Health Institute, Johannesburg, South Africa; 2 Division of Epidemiology and Biostatistics, School of Public Health, Faculty of Health Sciences, University of the Witwatersrand, Johannesburg, South Africa; 3 Mopani DCST, Department of Health, Limpopo Province, Giyani, South Africa; 4 School of Public Health & Family Medicine, University of Cape Town, Cape Town, South Africa; 5 Division of Infectious Diseases & HIV Medicine, Department of Medicine, University of Cape Town, Cape Town, South Africa; 6 Department of Medical Microbiology, School of Public Health and Primary Care (CAPHRI), Maastricht University Medical Centre (MUMC+), Maastricht, The Netherlands; Aga Khan University, KENYA

## Abstract

South Africa has implemented a community-based HIV programme (CBHP) in its primary healthcare (PHC) re-engineering strategy that aims to improve public healthcare delivery. This CBHP is delivered by ward-based outreach teams (WBOTs); provision of community HIV services comprises an important component of this programme. We conducted an exploratory study to determine the facilitators and barriers to successful implementation of this CBHP in rural Mopani District, South Africa. Focus group discussions were conducted with the community health workers (CHWs) and PHC nurses; participant interviews were conducted with community members who access these health services, community leaders, and social workers. We conducted a thematic content analysis and based on the key themes reported, we identified the Consolidated Framework for Implementation Research, consisting of five domains, as the most appropriate model to interpret our findings. First, in terms of intervention characteristics, community members generally valued the HIV services provided, but the variable needs impacted on programme implementation. Outer setting challenges include inability to meet the need of patients as a result of stigma, non-disclosure of HIV status and social factors. In terms of the inner setting, CHWs were grateful for the equipment and training received but expressed the need for better support of management and the provision of additional resources. With regard to characteristics of the implementers, the CHWs expressed the desire for further training despite reporting having sufficient knowledge to conduct their HIV work. Finally, in terms of the implementation process, the importance of relationship building between CHWs and community members was emphasised. In conclusion, these data underline the positive receipt and potential of the CBHP in this rural district and identify areas to further strengthen the programme. The success and sustainability of the CBHP requires ongoing commitment of resources, training, supervision, and organisational support in order to operate effectively and efficiently.

## Background

Community health programmes can improve public healthcare delivery, particularly in underserved settings [1]. There is a large body of evidence that suggests that community health programmes can improve access to healthcare in the communities that they serve [2–4]. Several low-and-middle income countries (LMICs) including Brazil, Ethiopia, Bangladesh, Malawi and Nepal have successfully implemented community programmes with community health workers (CHWs) at scale [5]. These countries have shown substantial gain in their maternal and child health and malaria programmes [5]. There is to our knowledge, limited information available regarding the provision of Human Immunodeficiency Virus (HIV) services at a community level.

South Africa has included a community health programme in its primary healthcare (PHC) re-engineering strategy, which was introduced by the National Department of Health (NDoH) in 2012 [6]. This community health programme is delivered by ward-based outreach teams (WBOTs) led by a team leader, who is usually a professional nurse. Each team consists of up to six CHWs, and is allocated 250 to 400 households to support [7]. CHWs play an integral role in promoting good health and preventing ill health by improving access to quality care at the community level. Provision of community HIV services comprise an important component of this programme. As such, they function as a crucial link between community members and the PHC system [5].

CHWs contribute to the HIV programme in South Africa by: 1) provision of information to educate individuals on how to prevent transmission of HIV infection; 2) identification of individuals at risk for HIV infection that should be tested for HIV; 3) provision of adherence support and tracing individuals with missed appointments and defaulters to improve retention in care; and 4) early identification of individuals with deteriorating health while on antiretroviral therapy (ART) to reduce further morbidity and mortality [8].

We recently assessed the fidelity of the community-based HIV programme (CBHP) as implemented by the CHWs in Limpopo province, South Africa. The findings show that there is good reach of the programme as well as appreciation from the community which they serve. However, we also showed that there was room for improvement to enhance impact of the programme. Community programmes interface with health systems and community systems, which require optimal integration on various levels. These relationships are complex and context-specific. Given this dynamic context, barriers and challenges to implementation may arise at different levels of healthcare delivery [9]. We therefore aimed to explore and better understand the facilitators and barriers that affect the CBHP implementation.

To our knowledge, there has been no formal exploratory assessment of the CBHP in Limpopo Province, South Africa. The aim of this study was to explore barriers and facilitators to the successful implementation of the CBHP to produce actionable findings to improve the programme.

## Methods

### Study setting

The study was conducted in the Greater Giyani and Greater Letaba sub-districts of Mopani District, Limpopo province, South Africa from January 2016 through August 2016. The communities in these sub-districts were similar in terms of socio-economic factors, but small local differences in ethnicity, cultural beliefs, and health practices are present. The CBHP was introduced and rolled-out from 2013. As of mid-year 2016, there were 149 active WBOTs covering 123 of the 125 (98%) wards in the districts. These support 222 261 registered households (75%) out of 296 321 households in the district [10]. The CHWs are employed by the NDoH, but are remunerated and managed by non-profit organisations (NPOs). A South African non-government organisation (NGO), Anova Health Institute provides technical support to the HIV programme in Mopani district, Limpopo.

### Study design and participants

We conducted an exploratory study to determine the facilitators and barriers to implementation of the CBHP. We used a comprehensive approach which entailed interviewing various stakeholders to obtain an all-inclusive viewpoint. This study was a part of a larger study conducted in twelve wards with a similar size catchment population, each served by one WBOT. The study sample includes a convenient sample (depending on availability of individuals at the time of three attempted visits) of community members, CHWs, team leaders, facility staff, community leaders, and social workers that reside in the twelve wards (Table 1). Individuals had to be adult (>18 years) to participate in the study and informed consent was obtained before conducting the interviews. Interviews were conducted by research staff, that had not been involved in programme implementation, as follows: 38 with community members, 9 with team leaders, 21 with community leaders, and 10 with social workers. Focus group discussions (FGDs) were conducted as follows: 11 with CHWs and 12 with facility nurses. This was sufficient to reach saturation based on the responses received. The private interviews were conducted and audio recorded in the participant’s home language (Tsonga or Sotho). The audio recorded interviews were translated into English and back-translated to ensure accuracy and consistency. This was done before data analysis by the research staff who were fluent in the relevant languages.

**Table 1:** Summary of study participants.

Target group approached to participate	Inclusion criteria	Interview type
Community	Household members aged 18 years and older.	In-depth interviews
CHWs	The CHWs that provide health services to the community in the 12 wards	Focus group discussions
Facility nurses	Facility nurses that are employed in the 12 wards.	Focus group discussions
CHW team leaders	Team leaders of the CHWs in the 12 wards.	In-depth interviews
Social workers	Social workers that provide services and support to the community in the 12 wards	In-depth interviews
Community leaders	Community leaders in the 12 wards	In-depth interviews

### Ethical approval

Written informed consent was obtained from all study participants. Ethical approval was obtained from the Human Research Ethics Committee at the University of Witwatersrand, Johannesburg, South Africa (Reference number: M1611111) as well as the Limpopo Provincial Health Research Committee.

### Data analysis

**Fig 1:**
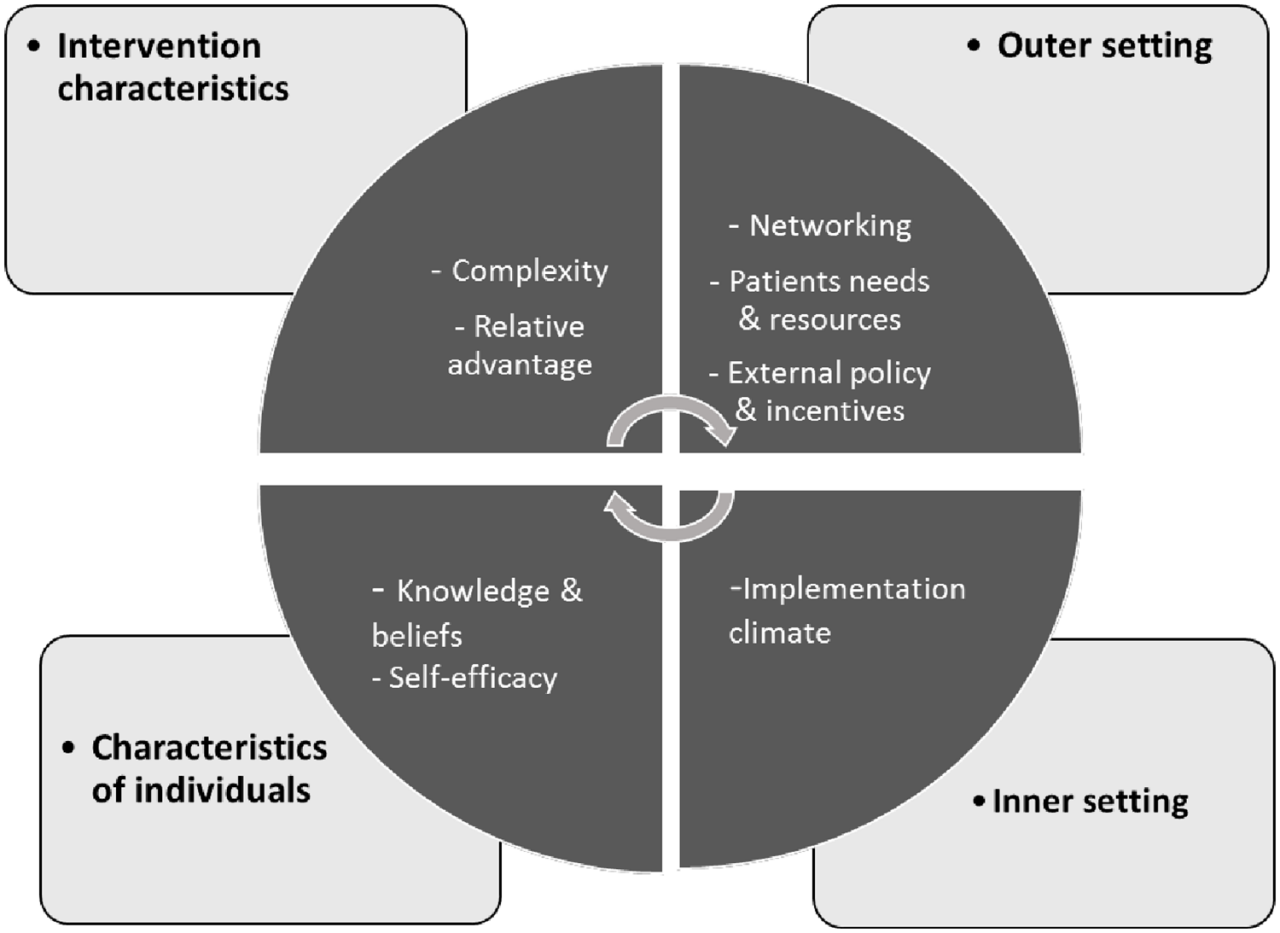
Consolidated Framework for Implementation Research (CFIR)–Adapted from Damschroder et al. [12].

The interviews were transcribed and imported into NVivo qualitative data analysis software [11]. Thematic analysis was used to analyse the data from the participant interviews and FGDs. Two of the authors (NN and GAJ) inductively generated codes, sub-codes, and broader themes. Inter-rater reliability was ensured through the continuous comparing of codes between the coders. In the final stages of inductive coding, the number of codes and themes were further re-arranged and reduced to formulate the final themes. Following thematic content analysis, based on the final themes reported by participants in this study, we identified the Consolidated Framework for Implementation Research (CFIR) as the most suitable model to provide a theoretical basis to interpret the findings. The CFIR is a conceptual framework developed to guide the systematic assessment of different implementation contexts to identify key factors that may influence the implementation and effectiveness of a given intervention [12]. The CFIR consists of five major domains that may affect implementation including: 1) intervention characteristics; 2) outer setting; 3) inner setting; 4) characteristics of individuals; and 5) the implementation process (Fig 1). Definitions of the five domains are further detailed in the results section. Within each domain we assessed key factors or constructs of the intervention that influence implementation [12].

## Results

Findings show that the CBHP was well received and there was an appreciation of the services provided by the CHWs. The CHWs reported to be very self-motivated and had adequate knowledge to impart to the community. This served as a facilitator to programme implementation. However, as with any intervention, barriers do exist which constrain programme implementation. The five CFIR domains and constructs of the CBHP are discussed below, detailing the way in which factors facilitate or constrain them and thus the implementation of the programme (Table 2).

**Table 2:** Summary of CFIR domains, constructs, definitions and determining factors identified in this study.

Domain	Construct *(Sub-construct)*	Definition	Factors affecting construct
**1. Intervention characteristics**	**Complexity**	Perceived difficulty of implementation reflected by duration, scope, radicalness, disruptiveness, centrality and intricacy and number of steps required to implement	Number of services required per patient
			Number of households to be visited
			Number of CHWs deployed in the community
**2. Outer setting**	**Networking**	The degree to which an organisation is networked with other external organisations	Formal: Lack of support from NGOs, NPO’s, NDoH, OPMs and social workers
			Informal: Support or lack thereof from community, community leaders, and traditional healers
			Inadequate integration of HIV programme into the facilities
	**Patient needs and resources**	The extent to which patient needs, as well as barriers and facilitators to meet those needs, are accurately known and prioritised by the organisation	Denial and fear of stigma
			Poverty
	**External policy and incentives**	Broad constructs that encompass external strategies to spread interventions, including policy and regulations, external mandates, recommendations and guidelines, pay-for-performance, collaboratives, and public or benchmark reporting	Lack of a formal programme
			Unclear CHW reporting structures
			High workload
			Inadequate stipends
			Undefined roles of CHWs
			CHWs find their work satisfying due to the positive impact on communities.
**3. Inner setting**	**Implementation climate: *Compatibility***	Compatibility refers to the degree of fit between the value attached to the intervention and the norms and perceived risks of individuals, as well as existing workflows and systems	Unacceptance of CHWs in the community
			Clear processes in place for defaulter tracing
			Lack of involvement and role clarification of social workers
			Poor integration into the PHC system in terms of existing workflows
	**Implementation climate: *Readiness for implementation***	The tangible and immediate indicators of the organisation’s commitment to its decision to implement an intervention. This consists of three sub-categories i.e. leadership engagement, available resources, and access to information and knowledge	Lack of involvement of OPMs with CHWs and team leaders
			Desire for ongoing training
			Inadequate resources and infrastructure (office space)
**4. Characteristics of individuals**	**Knowledge and beliefs**	Individuals’ attitudes toward and value placed on the intervention, as well as familiarity with facts, truths, and principles related to the intervention	Motivated CHWs
			Wealth of knowledge of CHWs
	**Self-efficacy**	Individual belief in their own capabilities to execute courses of action to achieve implementation goals	Adequate knowledge and skills
			Good initial training
			Desire for ongoing training
**5. Implementation process**	**Engaging**	Attracting and involving appropriate individuals in the implementation and the use of the intervention through a combined strategy of social marketing, education, role modelling, training, and other similar activities	Lack of relationship building with the community and traditional healers

### 1. The intervention characteristics

Intervention characteristics refer to the features of the intervention, and includes the constructs complexity and relative advantage in the CFIR [12].

Complexity is defined as the perceived difficulty of implementation of the programme by its implementers [13, 14]. An important factor related to the complexity of implementing the programme was the highly varying needs of individual patients. While some patients only require brief visits to ensure they are managing their ART adherence appropriately, others require significant amounts of assistance due to their poor states of health. This sometimes resulted in CHWs spending a large amount of time at each household, and being unable to achieve their daily targets. Another factor affecting intervention complexity is the large number of individuals to be visited by the CHWs and the limited number of CHWs deployed in the community, which also results in CHWs being unable to achieve their daily targets.

*“Sometimes we find out that a person is staying alone we are able to clean for him/her and we even make soft porridge for the person to eat*. *Sometimes we are able to wash their clothes so that they can be clean*.*”*

[CHW1]

*“Our work is too much*. *I sometimes tell myself that today I should cover more households as possible*. *The NGO coordinator does not mind to phone you when you are at work and ask you to attend to a patient far from where you are currently working*.“

[CHW2]

Relative advantage refers to the stakeholders perception of the advantage of implementing the intervention versus an alternate intervention or no intervention [15]. Overall community members appeared to value the services and were open to the services provided by the CHWs. Individuals expressed the relative advantage of the programme on an individual, community, and facility level, based on their interactions with the CHWs. On an individual level a clear advantage of the programme discussed by the community members was the direct assistance provided to them by CHWs, such as education on health-related issues, reminding individuals to collect their medication and performing chores for sick individuals such as cooking, cleaning, and fetching water.

*“They do their job very well*. *They help us with lot of things even things that we thought they are not possible to happen*.*”*

[Community member1]

“We really appreciate their work. People talk positive about the work of CHW’s”

[Community member2]

Community members also felt comfortable enough to disclose medical information, including their HIV status with them. This makes it easier for CHWs to refer patients to their nearest facility for testing and treatment.

*“What I like about visiting households*, *you find that a person is sick and he or she is scared to tell her family but when we get there the person is able to disclose to us*.*”*

[CHW3]

CHWs perceived their activities as beneficial to the community and this served as a means of motivation for them to remain in the programme, despite challenges.

*“We do have something rewarding*, *because when we find someone sick we are able to refer the person to the clinic*, *so he or she can receive suitable treatment*.*”*

[CHW4]

CHWs also reported that community members are receptive to health education about HIV and other chronic illnesses such as diabetes and hypertension.

*“Some people don’t know their HIV status*, *when we are teaching we advise them to go to the clinic to test*, *because we don’t carry anything to test*. *We see it significant because when we done teaching they go to the clinic to test*.*”*

[CHW5]

*“People see how important we are in the community*, *because we teach them about HIV and AIDS*. *We tell them that HIV does not kill*. *We advise them that when they test positive they should also check their CD4count so that if it is too low they can take treatment*.*”*

[CHW6]

On the community level, most community members interviewed were of the opinion that the programme has a positive impact on the community.

*“They have a good relationship with us*, *because they remind us with lot of things and they teach us things that we don’t know*. *Even on things that we don’t know that’s why I say they are good people*.*”*

[Community member3]

In addition, community leaders mostly expressed their gratitude for the contribution that CHWs make in the community, especially in the identification of at-risk individuals for HIV testing and adherence support. They appeared to be very supportive of CHWs and the services they provide.

*“Yes they are useful because if we can look back people used to die a lot because no one knew that they were sick*, *and death would occur at any time*. *Since they have been working in the community we are experiencing less death*. *Death does not occur like it used to in the beginning*.*”*

[Community leader1]

At a facility level, facility nurses and CHWs reported that the introduction of the community programme into PHC reengineering has improved workload at the facilities as a number of patients are being attended to at their homes. In contrast, the relative advantage of the community programme with CHWs was not universally accepted within the target communities, and CHWs noted that some community members refused to engage with them.

*“Some when they open their door they say if I knew it was you I wouldn’t have open the door*. *We are hoping that one day they will understand our significance*. *Hoping they will see that we are here to help them*.*”*

[CHW7]

### 2. The outer setting

The outer setting in the CFIR framework includes the economic, political, and social context within which a particular intervention is implemented [16]. In the context of this programme as implemented by CHWs, this refers to both formal and informal networks of support. In this regard we assessed the constructs networking, patient needs and resources, as well as external policies and incentives of the CFIR framework.

Networking is defined as the degree to which the implementers of the intervention in an organisation are acquainted with other external organisations [14]. Formal networks of support from the NDoH (the employer), NGOs, operational managers (OPMs) and social workers were reported to be limited. On a positive note, the CHWs more informal networks such as the community (the recipients) and community leaders largely played a role in supporting implementation of the programme.

*“We want the NGO and the department of health to support us on our work*. *We would like the NGO to come to our campaigns with food and other things*.

[CHW8]

*“PHC officers or OPMs at the clinic*. *They should not just buy in*, *they should buy in by supporting us and they should be involved in activities*. *There is no buy in*. *This program supports many different government departments*, *for example CHWs refer patients to social workers*. *Social workers when they are referred cases they do not do home visit to follow up because they have transport problem*.*”*

[Team leader1]

Patient needs and resources refers to the extent to which patient needs, as well as barriers and facilitators to meet those needs, are accurately known and prioritised by the implementers [17]. A few factors were noted to pose as barriers meeting patient’s needs. Denial and fear of stigma were among the most salient factors that emerged. As mentioned, community members mostly reported that they are being provided with health education and referrals to facilities for ART initiation and retention in care. However, patient’s denial of their HIV status, fear of stigma, and religious beliefs results in their avoidance of healthcare and support which renders CHWs referrals null and void. Patients sometimes also provide incorrect contact information at the clinic which makes it difficult for CHWs to locate them during defaulter tracing.

“Another challenge we have is people who are HIV positive, when we advise them to use condoms they refuse. They have unprotected sex. I don’t understand why they continue, maybe it’s because they are in denial”

[CHW9]

*“We find it difficult to get list of defaulters*. *In the area I work I had a case of someone who wrote wrong address and wrong age when registering a file*. *We found out later after looking for her that she lied*.*”*

[CHW10]

Poverty also emerged as a critical barrier. The very limited resources available to some patients meant that CHWs were unable to provide the level of care necessary to help patients recover or manage their illnesses appropriately. Examples of this include the lack of money for transport for patients to access facilities as well as deteriorating health of patients which pose barriers to patients accessing care and collecting their medication. Some community members do not have food to take with their medication. CHWs reported that they therefore distribute medication to patients at their households if patients are unable to visit their nearest facility.

*“There are some people who fail to go the clinic due to not having money for transport*. *Some become defaulters because of not having money for transport*.*”*

[CHW11]

*“Another challenge you find that a person stays alone and he uses a wheelchair*. *When the person has to come for treatment he has to find someone who can push him to the clinic*.*”*

[CHW12]

*“The most challenging thing we visit households that are poor and they do not have food to eat in order to take treatment*. *The reason people become weak is the result of taking pills without eating first*, *this is likely to create other sicknesses*.*”*

[CHW13]

External policies and incentives encompass external strategies to spread interventions, including policy and regulations, external mandates, recommendations and guidelines, pay-for-performance, collaborative efforts, and public or benchmark reporting [18].

Guidelines are available which detail CHW activities and roles. However, there were several suggestions from WBOT team leaders for WBOTs to be formalised by integrating them fully into the NDoH. This is an important challenge and was linked to a high degree of uncertainty surrounding the roles of CHWs and their reporting structures. Many CHWs appear to be undertaking roles that are not in their line of duty such as cooking and fetching water for community members.

*“In situations that we have to cook for a person in order to take treatment*, *it can make us not to reach the targeted number of households that we need to visit for that day*. *Sometimes we try to sacrifice but we fail*.*”*

[CHW14]

In addition, WBOT team leaders are professional nurses employed by the NDoH, which poses further challenges in terms of their workloads and reporting structures.

*“We are currently working for an NPO and PHC re-engineering program*. *Every month end they both require a report*. *We combine our work and submit our work to both programs*. *I think we need to know who we basically working for*.*”*

[CHW15]

*“Truly speaking the workload is too much*. *At the beginning I tried to balance two local areas and I realised that it is too much for me*. *I had to leave one and concentrate on the other*.*”*

[Team leader2]

In terms of incentives, there were also shared opinions by the CHWs, their team leaders, and community leaders that CHWs should be given increased stipends. The minimal stipends result in CHWs feeling demotivated to perform their duties. While the low stipends were viewed as problematic, CHWs reported finding their work satisfying due to the positive impacts they were able to make within their communities.

*“The first thing is the stipend*, *I am not saying this means that they have raised complaints with us*, *it’s not a matter of complaints but we know how much they get from the programme*. *At least that money should be increased in order to motivate them*.*”*

[Community leader2]

### 3. Inner setting

The inner setting in the CFIR framework relates to the structural characteristics and culture of the environment that may influence implementation [12]. The key CFIR construct relevant to inner setting in our study was the implementation climate. Implementation climate refers to the absorptive capacity for change, shared receptivity of involved individuals to an intervention [14], and the extent to which use of that intervention will be rewarded, supported, and expected within their organisation [19]. In light of this we assessed the sub-constructs compatibility and readiness for implementation of CFIR framework.

Compatibility refers to the degree of fit between the value attached to the intervention and the norms and perceived risks of individuals, as well as existing workflows and systems [12]. CHWs reported that they were not always welcomed by community members, and as a result sometimes struggled to perform household visits and were put in danger in terms of health and physical safety.

*“Some of the households that we used to visit they would chase us and even swear at us that we must go away*.*”*

[CHW16]

*“We are challenged because we do not have things to protect us from infection when we visit households*, *things such as masks*. *There are houses that we need to help but we find it difficult because we do not have a kit to protect ourselves*, *we do not have hand gloves and aprons to use when assisting patients*.*”*

[CHW17]

In terms of the programme fitting in with existing workflows and systems, a positive finding was that most CHWs reported that processes for defaulter tracing were clear and functional.

*“It is not a problem to get list of defaulters*, *data captures or nurses they give us the list*, *then we start working*. *We always get the list on time*.*”*

[CHW18]

A prominent challenge with regard to the CHWs integrating with existing workflows, is the insufficient involvement of social workers in the community and referrals to social workers not being dealt with. Social worker’s roles may also may require clarification in the community as some community members were reported to expect the social workers to assist with health-related issues.

*“I think the social workers should attend community meetings in order to speak to the community about their job roles*. *A lot of people don’t know where to report their problems but if the social workers address them they would be able to go the social workers*. *When we fail to help people they end up not trusting us*.*”*

[CHW19]

*“People in this community come to me as a social worker and they are expecting me to visit them in their households but I am not trained to deal with an ill person and I don’t even know where I should start*.*”*

[Social worker1]

Readiness for implementation refers to leadership engagement. This consists of the commitment, involvement and accountability of leaders and managers, available resources, and access to information and knowledge [12]. Several factors were prominent in affecting readiness for implementation. Firstly, CHWs and team leaders expressed their desire for more involvement from their superiors and a stronger support system. Secondly, in terms of available resources and access to information and knowledge, CHWs are grateful for the equipment (blood pressure machines and weight scales) and training received. However, other resources such as stationery and telephones are relatively limited and there is a need for the programme to be better supported and resourced in its entirety. Thirdly, CHWs expressed the desire for ongoing training from team leaders and NGOs in order to keep abreast with current HIV-related knowledge. Finally, there was also a consensus around the need for more space for the CBHP to operate from as the infrastructure within the facilities where CHWs work are limited.

*“This thing of giving us BPs test it seems like you have done a great job*. *Because now they know that we have BP test and weight scales*. *It seems like they like it because when we pass they call us to come and test them even those who were not interested*. *It seems like it is a job well done*.*”*

[CHW20]

*“We don’t have resources and sometimes we have to contribute money to buy stationery in order for us to be able to do the work*. *There is no stationery*, *no transport*, *no funds available*, *and even the mode of communication is so difficult because we don’t have anything*. *We are just sacrificing our own to render the services to our community*.*”*

[CHW21]

*“Our infrastructure is very small*, *there is no office space for them*, *and we just put them next to the sluice room which is not conducive*. *Even when we are holding the meetings we use the postnatal*, *what will happen when we have deliveries*? *There is no place where we can place them*.*”*

[Facility nurse1]

### 4. Characteristics of individuals

Organisations are composed of individuals (implementers) that are responsible for and thus influence implementation of an intervention [13]. We examined the constructs knowledge and beliefs and self-efficacy of the CFIR framework.

Knowledge and beliefs refer to individual’s (implementer’s) attitudes toward and value placed on the intervention, as well as familiarity with facts, truths, and principles related to the intervention [20]. Most CHWs seemed highly motivated by their work as it is has a large impact on the health of the community which they serve.

*“We do have something rewarding*, *because when we find someone sick we are able to refer the person to the clinic*, *so he or she can receive suitable treatment*.*”*

[CHW22]

The CHWs also serve as a means of monitoring the health of the community they serve, and as a result they have a wealth of knowledge regarding the number of vulnerable patients in the community and their health needs. CHWs report to have adequate knowledge and skills to conduct their HIV-related work.

*“What I can say in relation to experiences is that by the time we started working with the CHW’s they are able to manage our chronic clients and bedridden clients at home*. *Before they started doing this it was so difficult for us because they are the ones who are identifying problems*.*”*

[Facility nurse2]

Self-efficacy refers to implementer’s belief in their own capabilities to execute courses of action to achieve implementation goals [21]. We assessed CHWs belief in their own capabilities to conduct the implementation processes within their context. They found the initial trainings useful but also expressed the desire for additional training and new knowledge.

*“After the trainings that we got we knew and understood what we are supposed to do when we visit households*. *It equipped us with enough information on how to work with people who are sick*. *The training really helped us*.*”*

[CHW23]

### 5. Implementation process

The implementation process in the CFIR framework requires individual and organisational use of the intervention as prescribed. Individuals such as the implementers play a role in promoting the intervention and this may be influenced by the inner and outer setting [16]. In addition, activities to involve the organisational use of the intervention require involving relevant stakeholders to ensure optimal organisational change. In this light we assessed the construct engaging of the CFIR framework.

Engaging involves attracting and involving appropriate individuals in the implementation and use of the intervention through a combined strategy of social marketing, education, role modelling, training, and other similar activities [22]. An important area in which engaging played a role within the programme was the behaviour of patients in defaulting treatment taking. The CHWs express the importance of relationship building in order to try to mitigate these challenges.

*“Another thing is to be close with them and talk about the importance of taking treatment*, *while talking to them they will tell us the reason why they do not go to the clinic and take their treatment*. *When we have spoken to them they will be able to come to the clinic and take their treatment in order to continue*.*”*

[CHW24]

With regard to initiatives undertaken, it was reported that several campaigns and trainings were held with champions who drive the implementation, including CHWs and team leaders. Campaigns were also held with community members. External change agents were engaged during the implementation process for buy-in and support. These include individuals affiliated with an outside organisation that support the intervention such as NGOs and NPOs. Individuals who have a great influence on the outcome of the intervention such as community leaders and traditional healers were also engaged. Traditional healers seem to have a substantial influence of healthcare preferences of some community members, and some participants indicated that they would rather go to their traditional healers for healthcare and medical advice as opposed to the local clinic.

*“There are people who are sick who only go to traditional healers*. *This people think that they need ancestral ceremony they will be healed*. *People like this do not even come to the clinic*.*”*

[CHW25]

Some traditional healers are knowledgeable about the importance of primary healthcare and acknowledged the CHWs and the services they provide, while others pose a challenge to the programme as they negatively influence the health-seeking behaviour of the community, making ongoing engagement by CHWs an essential component of the success of programme implementation.

*“I wanted to add on traditional healers*, *the traditional healers that we have in the community they are educated; it is only the new traditional healers that are not educated*. *The traditional healers that are not educated maybe they are the ones who can cause problems*.*”*

[CHW26]

## Discussion

In this study we used the CFIR framework for identifying and understanding the factors that influence the implementation of the CBHP in a positive and negative manner. The key barriers and facilitators to implementation were examined systematically across the CFIR domains and constructs. This enabled us to generate actionable findings that can be used to improve implementation of the programme.

In terms of the intervention’s characteristics, challenges around the complexity of implementation can be addressed by employing additional CHWs, re-assessing CHW daily targets and staff workload, and provision of transport in order to increase the number of household visits in a day.

With regard to relative advantage findings show that the community value the services and were open to the services provided by the CHWs. Community leaders expressed their gratitude for the contribution that CHWs make in the community, with regard to health education and adherence support. Facility nurses also reported that the introduction of the community programme into PHC reengineering has improved workload at the facilities. However, there is room for improvement with regard to the acceptance of the programme. This can be addressed by undertaking community interventions such as campaigns to improve the community’s and traditional healer’s knowledge and acceptance of CHWs.

The success of the community programme depends on networking in order to gain buy-in, support, and involvement of the NDoH, PHC staff, and NGOs. It is therefore imperative to address the networking gaps by improving communication channels and creating a feedback loop to ensure that all stakeholders are appropriately involved and play their respective roles. Interventions such as campaigns to improve health education and stigma-related issues should be considered to address patient needs and resources. Strengthening linkages to social workers is needed to address the needs of patients and improve their well-being. In addition, the involvement of the NDoH and NGOs in the provision of food parcels and transport to healthcare facilities to poverty-stricken patients will also assist in alleviating the community resource-related problems.

The external policy and incentives barriers can be circumvented by designing and implementing formal NDoH policies which clearly stipulate roles of CHWs. In addition, the policy should ensure that the team leaders (professional nurses) are solely assigned WBOT duties. CHWs should be provided with an increased stipend to allow them to support their families and to provide motivation for them sustain their role the HIV programme.

Strengthening linkages between the community and social workers as well as clarification of social worker roles is needed to address the compatibility of the programme with existing workflows. This will in turn address the needs of patients and improve their well-being. Additional resource allocation and the improvement of existing infrastructures is needed to improve the readiness for implementation.

In terms of knowledge and beliefs and self-efficacy, CHWs had adequate knowledge to conduct their HIV-related work and were very enthusiastic to be a part of the programme. CHWs found the initial training very useful which equipped them to conduct their work. Ongoing training should be provided to keep CHWs abreast with current HIV-related knowledge which will, in turn, improve their working environment. Only some traditional healers are knowledgeable about the importance of primary healthcare and acknowledged the CHWs and the services they provide. Therefore it is imperative to engage with them and to involve them in campaigns and regular meetings. This will provide education and knowledge on how to help sick community members and when to make necessary referrals to the facilities.

These findings are corroborated by a recent study consisting of four case studies in Lesotho, Mozambique, Swaziland, and South Africa. Findings show that the key facilitators to implementation of the community based programmes include recognition of the CHWs by the government, standardised training provided through the government, and the existence of a proper organisational structure dedicated to healthcare initiatives [23]. Suggestions for improvement of the community-based programmes include provision of adequate resources to support and sustain CHW services, hiring of more CHWs with better incentives and career prospects, developing better links with health facilities, and providing political stability and continuity for community-based HIV services [23]. Findings from our study in terms of the barriers to successful implementation of community-based programmes are further correlated with findings form other studies undertaken in LMICs [24–26].

The comprehensive exploratory approach of this study allowed for a well-defined study, including perspectives from several stakeholders. Participant interviews and FGDs were ideal for assessing factors surrounding the complexities of implementing an intervention. The limitations of this study include the context-specific nature of the results, which should be considered when applying the findings to other parts of South Africa. The convenience sampling of the community members may have introduced bias and the findings should not be taken to be representative of the population. In addition, the employers of the CHWs were not interviewed as they were unavailable due to operational reasons. As with all research, another limitation is the possibility of respondents providing desirable answers as the researchers were affiliated with the Anova Health Institute.

## Conclusions

This study provides actionable findings of factors that facilitate and constrain the successful and sustainable implementation of the CBHP. The reported barriers and challenges can be used to inform stakeholders on how to improve implementation of the programme. The key factors that contribute to the success of this programme include relationship building with the community and other stakeholders to promote buy-in as well as proper integration of the programme at the facility-level. These facilitators can be expanded on, to further improve the CBHP and also be used in other areas with a similar contextual settings. Overarching barriers that can be improved upon include resource limitations and inadequate integration into the PHC system and workflows. More specific factors for improvement include formalising the WBOTs and increasing the CHW stipends. This approach has a broad application which adds value to research. We encourage others to use this CFIR as it allows researchers to use a common approach and language to systematically and comprehensively assess multicomponent interventions. This will enable researchers to compare findings from similar contextual settings to guide implementation of similar interventions.

## Supporting information

S1 Information Transcript excerpts from an interview with a community leader. (PDF)

S2 Information Transcript excerpts from an interview with a community member. (PDF)

S3 Information Transcript excerpts from an interview with a social worker. (PDF)

S4 Information Transcript excerpts from an interview with a team leader. (PDF)

S5 Information Transcript excerpts from an interview with a CHWs. (PDF)

S6 Information Transcript excerpts from an interview with a facility nurses. (PDF)

S7 Information Interview guide CHWs. (PDF)

S8 Information Interview guide community. (PDF)

S9 Information Interview guide community leader. (PDF)

S10 Information Interview guide facility nurse. (PDF)

S11 Information Interview guide social worker. (PDF)

S12 Information Interview guide team leader. (PDF)
