# Supplementary material for: Qualitative assessment of facilitators and barriers to HIV programme implementation by community health workers in Mopani district, South Africa
Source: PLoS One. 2018 Aug 30;13(8):e0203081. doi: 10.1371/journal.pone.0203081 (PMC6117027; doi:10.1371/journal.pone.0203081)
Supplement: S1 Information — (PDF) [file pone.0203081.s001.pdf]

Date conducted: 01/05/2016 Interviewee type: IDI

Date transcribed: 27/06/2016 Site: Facility 4

Interviewer: NZ

Interviewer : Do you have interaction with the community health workers?

Interviewee : We interact with them regularly through the clinic as I am the chairperson of the clinic committee; we always have interactions with them. We know their roles and responsibilities.

Interviewer : What are your experiences with them?

Interviewee : Our experience interacting with them is that we found that they are playing a major role in our community, but at the same time there are challenges they are experiencing, especially the issue of transport. They have to walk around the community the whole day without transport, and when people in the villages have to be assisted it is also a challenge.

Interviewer : What do you think are the roles of community health workers?

Interviewee : I think their roles and responsibilities are door to door visits as they always do and to check people who are sick and render assistance. We are gathering that these people are helpful because most of these people they are assisting live alone in their household and there is no one to assist them. So the community workers always reach them, they reach places where the clinic cannot reach.

Interviewer : Do you think the community health workers are useful or not useful?

Interviewee : They are very useful to the community because the clinic would not be able to reach these patients without community

health workers as they go door to door. Even the community members may not know exactly in which household there is a problem of health and stuff like that. The community health workers do inspections when they conduct their door to door visits on daily basis, and they know who is sick and how that person can be helped.

Interviewer : If there are challenges, for instance, the issue of transportation and other challenges, how can these issues be solved?

Interviewee : I think this problem needs funding from somewhere, maybe from the NGO's so that there is at least one vehicle on standby at the clinic so that when the community health workers encounter problems they can use that vehicle to assist. Not necessarily on the cases of door to door visits but when there are special cases in a sense that people have to get to the clinic urgently.

Interviewer : Let us move to our last part, I want to understand what are your roles and responsibilities as a community leader?

Interviewee : Our role as community leaders is that this program is community based, if there are community meetings it is our responsibility to highlight to the community members regarding the roles played by the community workers, and if there are challenges they must know who to contact in cases where there is someone who is sick and cannot be contacted. So they actually know where to go and what to do

Interviewer : Let's go out a bit from the work done by the community health workers, in general what are your roles or responsibility as a community leader in this village?

Interviewee : I am the chairperson of the clinic committee and my role is to give information to the community regarding these programs so that people know what to do if there are challenges

Interviewer : As a community leader what are the challenges you face?

Interviewee : The challenges we face, especially with the case of the community health care workers, there are people who want to participate in this program but the issue of stipend is a problem because when you look at the role played by the community workers; they work the whole day Monday to Friday, and you look at the money they get from the program is too little. We also recommend that the community health care workers be hired on fulltime bases. There should be people who work on weekends because health doesn't not only work on Monday to Friday. Even on weekends people do need assistance. This can only happen if their number is increased so that they are people who are on standby who can assist on weekends

Interviewer : This is my last point, how does your role as a community leader affect the roles of the CHW's?

Interviewee : When they go around the community they know that it is not only the clinic that is monitoring them, we are also looking at their role in the community. With a good relationship we have it makes things easier

Interviewer : In short, before we close the interview, how do you think we can improve the work of CHW's?

Interviewee : The first thing is the stipend, I am not saying this means that they have raised complaints with us, it's not a matter of complaints but we know how much they get from the program. At least that money should be increased in order to motivate them. Secondly, if we can have some kind of transport on standby that would add a great value on this program

Interviewer : I think we have covered everything, thanks a lot.
