# Supplementary material for: Qualitative assessment of facilitators and barriers to HIV programme implementation by community health workers in Mopani district, South Africa
Source: PLoS One. 2018 Aug 30;13(8):e0203081. doi: 10.1371/journal.pone.0203081 (PMC6117027; doi:10.1371/journal.pone.0203081)
Supplement: S2 Information — (PDF) [file pone.0203081.s002.pdf]

Date conducted: 12/09/2016 Interviewee type: IDI

Date transcribed: 15/10/2016 Site: Facility 7

Interviewer: NZ

Interviewer:	Were you visited by a CHW in the last 3 months?
Interviewee:	No, they have never visited my house.
Interviewer:	What role do you think CHWs have in your community?
Interviewee:	They visit people who are sick
Interviewer:	What can they do for you?
Interviewee:	The things that they do for others.
Interviewer:	What makes good CHW?
Interviewee:	Because they help those who are sick. I always see them visiting people.
Interviewer:	When you say they visit sick people were you told by someone?
Interviewee:	No. I see them visiting houses that there is someone sick.
Interviewer:	The people that the CHWs visit what Illness do they have?
Interviewee:	ahhh! That I don’t know. I think they visit anyone who is sick it does not matter what the sickness is. If they were to find me sick they will help me and they will also give me pills.
Interviewer:	Would you feel comfortable to share any health information with the CHWs?

Interviewee: Yes, when I am sick I will call them to tell them that I am sick; I know that they are the ones who help people who are sick.

Interviewer: Do you know your HIV status?

Interviewee: Yes I have tested.

Interviewer: If you were HIV positive, would you tell your family?

Interviewee: Yes, I would tell them so that when I die they would know what killed.

Interviewer: If you were HIV positive, would you tell the CHW?

Interviewee: Yes

Interviewer: Why?

Interviewee: I would tell them. I could even call them to come and help me. I could tell them because I would want to live.

Interviewer: If you were positive would you visit the clinic?

Interviewee: Yes I would.

Interviewer: What were your experiences at the clinic?

Interviewee: There is no problem. People get helped so I think they are treated well.

Interviewer: Do you think there is anything that could be improved at the clinic?

Interviewee: The number of nurses. Sometimes when you go the clinic you find two nurses working, some you find that are on lunch. The nurses that are on shift they have to attend every patient that has visited the clinic and the patients end up going home late.

Interviewer:	What do you think about having a support group for people taking ARVs in your community?
Interviewee:	I think people who are sick they hide. They would find it hard to attend the group but I would advise them to attend the group without mentioning that they are sick.
Interviewer:	What is the reason that makes people hide when they are sick?
Interviewee:	They hide because they are afraid that people will talk about them that they are sick. Long time ago people who were sick used to die but now the number has changed.
Interviewer:	Would you attend a group like this?
Interviewee:	Yes I would attend.
Interviewer:	Why?
Interviewee:	Because I will be attending with people who are sick.
Interviewer:	Have you ever been in contact with social workers?
Interviewee:	To do what?
Interviewer:	Anything?
Interviewee:	No I have never been.
Interviewer:	Have you ever visited a traditional healer?
Interviewee:	I only get help at church. I have never gone to traditional healers.
Interviewer:	What are the reasons you do not go to traditional healers?

Interviewee:	Because I get help at church I have never had a problem. I don't know in future but now I am helped at church. They give me things that help me.
Interviewer:	What do you think are the role of traditional healers in the community?
Interviewee:	I don't know how they work. If I had once visited them I would know what they do.
Interviewer:	Which church do you go to?
Interviewee:	ZCC.
Interviewer:	If given a choice, would you go to the clinic or traditional healer for health related problems?
Interviewee:	I would go to the clinic?
Interviewer:	Why
Interviewee:	Because I had never visited traditional healers before. I think I will get help there.
Interviewer:	Thank you for your time.
