# Supplementary material for: Qualitative assessment of facilitators and barriers to HIV programme implementation by community health workers in Mopani district, South Africa
Source: PLoS One. 2018 Aug 30;13(8):e0203081. doi: 10.1371/journal.pone.0203081 (PMC6117027; doi:10.1371/journal.pone.0203081)
Supplement: S3 Information — (PDF) [file pone.0203081.s003.pdf]

Date conducted: 08/06/2016 Interviewee type: IDI

Date transcribed: 20/09//2016 Site: Facility 5

Interviewer: NZ

Interviewer : Ok let us start with our discussion, it won't be too long, there is no wrong or right answer and please be free to speak about anything, either positive or negative. What are your experiences working with the CHW's?

Interviewee : So far I haven't noticed anything bad and most of the times I am not around but when they encounter something in my absentia they refer it to me when I come back. So far we have a good working relationship

Interviewer : You have mentioned that they refer some cases to you; what kind of cases they refer to you?

Interviewee : Many families around these villages are affected by poverty, and you find that in a family there is someone who is HIV positive and they don't have anything to eat and you find that I am not in the office, so they refer such cases. They only deal with the medical part of the patient and when they feel that there is a social aspect that when they involve me

Interviewer : When you intervene how do you do that?

Interviewee : After they have made a referral I will have to attend to that patient by making a visit and if the department have some food parcel I will give them but if they don’t I only refer them to SASA because they are the one who usually provide them with food parcels

Interviewer : As a social worker how is your impact in the community?

Interviewee : We provide psychological support and when someone is finding it difficult living with HIV, we give them support and provide them with counselling. But we are in a challenge of shortage of resources and sometimes we fail to meet the needs of the clients because we don’t have resources to cover their needs. As I have mentioned that the case of food parcels you find that the family is poor and you try to give them psychological support but the actual need is food and you can’t give them food because you don’t have parcels in the department.

Interviewer : I think you have raised important points here, can you please unpack it a bit more when you say you fail to meet the clients need because of a lack of resources,

Interviewee : We don’t have much resource; we don’t have resources to a point that in my office I am sitting on a list of more than 50 people who are in serious need of food parcels and some of them are being referred to me

by CHWs. We are told that the department doesn't have a budget for food parcels. They are referring them to us but there is nothing we can do

Interviewer : To get a clear understanding, what are you exactly referring to when you talk about resources?

Interviewee : Food parcels, don't have a toner, and sometimes you want to make a referral to the hospital but you cannot make that referral because you don't a simple thing like a toner to print, and you cannot even refer someone to go see a psychologist at the hospital or see a nurse

Interviewer : How is this affecting your work?

Interviewee : It is negatively affecting our work because the clients think that we don't want to help them, they don't understand what is happening in the office. It seems like I don't want to help them and that is not the case. You try by all your means and you have a list but you cannot give them what they need, and at the end of the day it seems like the social worker is not working whereas we are working but we don't have resources to support us.

Interviewer : You spoke about the issue of referrals; I just want to find out from you that how many cases they have referred to you?

Interviewee : I don't keep count, I differ from month to month, and sometimes the department would say they need referrals for food parcels and since I am working in three

villages, Maphalle, Tshawela, and Detshosini, I don't know everyone in these villages so the CHWs are the ones who go and identify these households. I don't have the exact number. When they work in the community and they find something they refer to me. I don't have specific numbers but they do refer

Interviewer : Yes I understand, what would be your estimate of referrals they have made to you?

Interviewee : I have two years on this job and I think they have made +- 100 referrals

Interviewer : Ok let us proceed; do the CHWs visit the clinic?

Interviewee : Yes they do

Interviewer : How often do they visit?

Interviewee : I am not sure I can't lie

Interviewer : Have you experienced any challenges with the CHWs

Interviewee : No I haven't. As I have said I don't know many people in the community and but they know each other. So if I have a case I ask them, sometimes they do help me when a case

Interviewer : When they refer clients to you what do they use?

Interviewee : Some use referral forms and some they don't. They just bring someone into the office and they tell you why they brought that person, but I have seen some referrals.

Interviewer : What do you think is the reason some don't bring referrals when some are giving you?

Interviewee : I think some are lazy to write I don't know because some do bring referrals and some don't. I don't know why I have never asked them

Interviewer : How do the CHWs affect your workload?

Interviewee : As I have said they are really helping us a lot; especially on cases of HIV/Aids because we are expected to report every month on HIV/Aids and when you don't have contact with them it mean you will have nothing to report about. So we ask them for cases and they refer them to us. For me they have a good impact on my workload because I am able to report at the end of the month.

Interviewer : You asked them to go out and look for people who are HIV positive?

Interviewee : They do refer cases every month so we use those cases. After they have referred the client we do home visits and obviously I will have to report because I have made a home visit since I was in contact with the client

Interviewer : Do you find the CHWs useful or not useful in the system?

Interviewee : They are useful,

Interviewer : Let me push you a bit, why do you think they are useful?

Interviewee : For us social workers and nurses, as well as the dietician we are based at the clinic and usually we don't

know what is happening in the house of the people. So we get the information through them, and we work at Maphalle but we don't know Maphalle, so they know Maphalle more than we do. If the CHWs were not there most of the people would die at home and we won't be aware of it, but because they are here the community feel freer to refer the clients to them than coming to us. So I think they are really helping us a lot, I don't know for other people but for me I really see their impact

Interviewer : Ok let me push this a bit more, you say they feel more comfortable to refer clients to CHWs than you guys, what do you think is the cause for that?

Interviewee : I think that people are not used to us, they are used to the CHWs because they are part of their community. As I have stated Maphalle is a very large area, for someone who stays at the RDP section it would be very difficult to come here to report a case. So it is better for them to report it to the CHWs and they will report it to us. They really doing their work, I don't know how much impact they make at the department of health but for me they are

Interviewer : So what you are saying is that they are more accessible or approachable than you guys?

Interviewee : Not really about approachable but they are available than we do, they are available

Interviewer : Ok what could the CHWs do for you?

Interviewee : I think what they are doing, referrals and stuff, it's ok for me because most of the things we do are very confidential. Once they have referred a case to me its ok I will take it from there. A referral is more than enough for me, I don't need anything else

Interviewer : Ok let me ask you few questions about your roles and experiences as a social worker in this area, we are about to finish and are only left with few questions as you said that you are rushing somewhere. What are your roles as a social worker in this area?

Interviewee : I have a lot of roles; I do foster care and I have more than 50 files for foster care, so the workload is too much. There are many children out there who are orphans and vulnerable.

Interviewer : Can I please understand how do you help those children?

Interviewee : When parents die we apply for foster care grants; we do our investigations and then take the case to the court. The magistrate will declare the child to be in need of care and protection and them I take their family to apply for a grant. They get R850 per child on monthly bases, so I have to make sure that it is really being used for the child because some of the foster parents don't really use the money on those children. So we keep on checking

them and visiting them. There are a lot of these cases and I am alone. So sometimes I take up to four months without visit them, but I try to check them usually in order for me to see if the children are really being take care of. Sometimes you find that a child is receiving foster care money but the child is not been look after, they don't have school uniform. So we keep in contact with the foster parents to make sure that the needs of these children are met.

We do family preservation cases, for instance, if the husband and a wife are having a misunderstanding at home or it's a mother and a daughter kind of a situation, we call them and have a session with them and try to make peace and make them to live in peace with one another. When you find a case of a husband and a wife who wants to separate and we have tried everything and they don't want to listen to us we then refer that case to the magistrate so they can continue there with their case of divorce.

We do group work with some of the staff here at the clinic, for HIV/Aids. We have a support group that we run on monthly bases for people who are living with HIV/Aids.

We do Monitoring of crèches and all NGO who are funded by the department. We go around monitoring

them once every quarter. I have 5 which are funded so I have to make sure that I monitor them.

If someone doesn't have a birth certificate we will have to help them with report writing so that the home affairs can give them certificates.

Social work is a profession of many phases, if someone comes and they have been raped and under age we represent them at the courts. That's what we do

Interviewer : How manageable do you find your job at the present moment?

Interviewee : What is that?

Interviewer : We want to find out about your workload

Interviewee : I do have too much workload and sometimes I have to take work home. We don't have many social workers here, we have a lot of workload and only few social workers and Limpopo is not employing social workers so we have to deal with everything and that's what I am expected to do

Interviewer : Do you cope with that or you struggling to cope

Interviewee : I try and sometimes I have to compromise my family time and take work home, for example, if I have to go the court sometimes I have to take my report home so that I do them overnight so I can send them the following day.

Interviewer : Ok we are almost finished, what I want to ask you is what are some of the challenges that you face as a social worker?

Interviewee : Resources is the main challenge, and we have a lot of workload and no one to assist. Resources covers everything and workload

Interviewer : Ok for our sake as we don't know which resources you are referring to,

Interviewee : Stationery, vehicle, office phone and that I have to use my personal phone to call clients, travelling to the other office just to print reports every time I need to print, making clients to wait maybe for a month before I can receive a vehicle in order for me to make a home visits,

Interviewer : In conclusion, what can be done to improve on these challenges?

Interviewee : Only if the department can respond to our needs. The department knows that we need these things; they know that we need a car and a computer in the office. Only the department can respond to what we need

Interviewer : So currently they are not responding?

Interviewee : They are not responding hence we are on strike. We are not working here in Limpopo; we just come to the office and sit

Interviewer : What are you striking for if I may ask?

Interviewee : Better working conditions, better salaries because we are on level 7 and we feel like it is not enough and we have lot of workload. We need employment of social workers because I think Limpopo is sitting on 500 and something unemployed social workers. That is what we are striking for. We just come in the office and we only do what we can and we are not doing things which are beyond our control. If the client comes and they need something that needs typing and I don't have a toner I will not go an extra mile and use my money, I can't do that. We have to sit in the office until the department do something

Interviewer : When did you start with the strike?

Interviewee : We are on week three now but last month we matched to Polokwane and the response they gave us was very negative so now it's a national strike and we are going to match again in Pretoria on the 19th of September. Up until then we said we are not going to do anything beyond what we can do. If we don't have resources we just tell the client we don't have what you want come back next time when we have what you want. Back then we used to compromise with our phone and everything, our monies and even our personal laptops, but now we are saying enough is enough we not going to do anything beyond what we can do

Interviewer : You spoke about level 7 and 9,

Interviewee : Yes it’s a pay level and we are on level 7 and somehow we are robed because we went to varsity with nurses and we did a four year degree including honours but we get paid on level 7 but the nurses are getting level 8 and we don’t have anything in our offices. So we are asking the department to give us level 9 or 10 but they are saying they can’t do that. That’s why we decided to strike. We want better pay and working conditions; some people are working in containers and at least I am based in the clinic I have water and I am in a safe place. Some people are in mobile containers and they don’t have water and electricity. In Matshwi clinic the social worker doesn’t even have a computer but she is expected to submit monthly reports which must be typed but which computer must she use? They are expecting her to deliver from her own resources. We are saying enough is enough

Interviewer : Ok I think I have asked almost everything, so as you said you have to rush, thank you for your time and views, we highly appreciated.
