# Supplementary material for: Qualitative assessment of facilitators and barriers to HIV programme implementation by community health workers in Mopani district, South Africa
Source: PLoS One. 2018 Aug 30;13(8):e0203081. doi: 10.1371/journal.pone.0203081 (PMC6117027; doi:10.1371/journal.pone.0203081)
Supplement: S4 Information — (PDF) [file pone.0203081.s004.pdf]

Date conducted: 19/05/2016016 Interviewee type: IDI

Date transcribed: 06/09/2016 Site: Facility 7

Interviewer: NZ

Interviewer : How often do you meet with the CHWs?

Interviewee : Twice per month; usually fourth night and month end, that’s when we meet.

Interviewer : What is discussed during the meetings with the CHWs?

Interviewee : Usually for the month end they bring back the reports and we give them the reports of the meetings, and we give them in-service training. And also if they have queries or problems that we didn’t attend to during the course of the two weeks that I didn’t see them, I attend to them.

Interviewer : So on the issue of reports, what do they report on?

Interviewee : Usually we have a format which they use to report have two reports, because in our project we have to attend five things; under five children and ANC postpartum , so they have to bring the report of all the visits they have done, and also report on how many pregnant women they have attended to until month end. So I have to compile a statistic of how many hours work they have visited.

Interviewer : So they give you a report once a month?

Interviewee : Yes once a month.

Interviewer : You spoke about the in-service training, what do you train them on?

Interviewee : Usually because we are attending on chronic patients, I teach them about diabetics and any chronic condition that they have to deal with like hypertension, because when they were in the NGO they were attending on HIV and TB, so that is what they know most. But because this project is broad they don't know the signs and symptoms of hypertension, diabetics and arthritis, so that is what I teach them about and to how to find out if the patient has hypertension. And we also teach them how to check a BP because they have the machines to do so.

Interviewer : I heard that they were trained before they start the work, didn't they teach about that?

Interviewee : No, this side they only learned about phase one, phase one is only TB and HIV, so Phase two includes Hypertension and diabetics, so they didn't do the phase two.

Interviewer : So do you have Phase three?

Interviewee : No we don't have phase 3, phase 3 is about Shiluvani sub-district, the CHWs still have to go through a one year six months training offered by the Government, but we don't know when it's going to happen.

Interviewer : Do you find the meetings useful?

Interviewee : Yes, a lot because if we don't have them there will be no two way communication between us and the CHWs because we don't have any means of communication, unless if I use my airtime and call the CHWs. But is best if I meet with them because we can also address

those issues which they came across when they were doing the home visits so that we can address them.

Interviewer : What could be done to make these meetings more useful?

Interviewee : Usually the CHWs stays far and they don’t earn so much, sometimes when I call them to come to the meetings you may find out the person we saw last month won’t be the person we going to see next month, because of the transport and money issues, they have to rotate, so they just gather the money and send it to someone. We got about 39 CHWs but you may find that there is that sometimes only 30 or 20 came because of the issue of transport money.

Interviewer : You mentioned that they don’t earn much, How much are they earning exactly?

Interviewee : R1500 stipend from their NGOs.

Interviewer : Can you tell us in detail what exactly do you discuss on your monthly meetings?

Interviewee : on our monthly meetings, usually the first thing I do is give them the reports of the meetings that I have attended, and secondly if there is an in-service training I will inform them about that, and after that I will conduct the in-service training after giving them the reports of the meetings. Then lastly I tell them to ask if they have any questions, like asking me what to do they have come across a situation where the children was not receiving grants or not well taken care of, then they will ask me what do they do in that kind of situation and then I will give them a way forward on which steps to follow. Then I will tell them to go to the clinic and find the Social worker to go and assist that family. And i

also tell them that they must go back and check if the Social Worker has attended to that child.

Interviewer : What are the main Questions does the CHWs ask you?

Interviewee : The first question they ask is about money, because they always ask when are they going to get paid ? And that they use their own money to travel and even to pay for some stationery if there is no enough stationery. And another thing they ask is about the project, they want to know where is the project taking them because they are still in the NGO but we use them as Government resources but they don't get paid for that. And it has been three years now and nothing is changing. So they want to be secured if they going to be employed as Government employees or not, but all those things I don't have the answers for them, but it is always the issue of money.

Interviewer : So do you think those questions they ask affect the way they work?

Interviewee : Yes a lot ,because we can't control them mostly, especially when we are not paying them, sometime they say today I am taking my child to school, I can't say no they can't go because I have work they must do, because I am not paying them. It affects a lot, or say today we are doing the NGO work, you say anything because the NGO pays them. And usually also from my side they work from 08:00-13:00, so from 14:00 to 16:00 they don't do the home visits. If there was money I would say to them please come to this project and work the whole day. So it does affect the work a lot.

Interviewer : You said you have 39 CHWs right?

Interviewee : Yes 39, including from other clinics, Senopela Clinic I have 16, but today we have 8. So is covering Ward 1, this side of Tzaneen and Ward 21 is that other side of the mountain.

Interviewer : What are some of the challenges reported to you by the CHWs?

Interviewee : The first one is the Manager, secondly is the NGO because they don't give them travel allowance, and when we do the workshops they don't have money to come and attend the workshop. The other thing is that I give them the monthly schedule but because they still under the NGO they won't come because the NGO want them as well.

Interviewer : How is your relationship with NGO?

Interviewee : From my side I don't have any problem with the NGO, because if I ask them that I need the CHWs they release them and they will come and work with us. But sometimes they have to send the stipend and tell them to come to the office and they will go without giving me the information they were supposed to give me. So sometimes when I pass the NGO's office and see the CHW they will tell me that they came to sign for the stipend.

Interviewer : So you said the other one is the NGO and the other one the Re-engineering, so how do they write the report?

Interviewee : When they fighting that they must report form one thing, So the NGO still don't want to use our reports , they say it is not the same, it not the same information from their register, they say the patients they have on their register are not the same as the ones the CHWs are

visiting. They are writing two reports actually, one for the NGOs and the other for Re-engineering, but for 1 patient.

Interviewer : So how do the CHWs feel about writing two reports?

Interviewee : Firstly when we started doing this they submitted the report for the Re-engineering to the NGO, then they said no we want other one not this one, so they feel left out, because usually they say you the CHWs you feel like you are promoted , so sometimes they feel demoralised .

Interviewer : So they are told that by who that they think they are promoted?

Interviewee : The Manager of the NGO.

Interviewer : Why does the Manager tell the CHWs that they think they are promoted?

Interviewee : There are politics inside the NGO, so sometimes I don't want to get myself involved in the NGO, because they have their own politics.

Interviewer : How do you personally feel about the way things are going with the NGO and the CHWs?

Interviewee : I just reassure the CHWs and tell them to work hard because we don't know where this project is taking us, I tell them that your Managers will always talk but let's just focus on our work. I just reassure them.

Interviewer How do you think we can improve to help with the CHW with the challenges they are facing?

Interviewee : I think if they get paid and maybe being absorbed by Government, at least it will be something and they will be happy.

Interviewer : Let's go back a bit, you mentioned that they are two challenges: the issue of money and the issue of NGOs, Are there any other challenges you didn't mention?

Interviewee : Yes there are, Inside the clinic when they refer the patients, let's say they went to the household and found the patient is in a critical condition, and they refer them to the clinic and the nurses don't want to be listen to the CHWs, they will be saying which school did they go to study that things like that, that is the challenge they are facing. But sometimes I sit down with the clinic manager and explain to them what knowledge do the CHWs have, the baby, the mother, women's department, at least now we are on the same line.

Interviewer : Do they tell the patients or someone else about who wrote about who wrote the referral letter for them?

Interviewee : They tell the patient.

Interviewer : How does the patient react after hearing that?

Interviewee : The patient will no longer trust the CHWs.

Interviewer : Do they help the patient at the clinic?

Interviewee : Yes they do help the patient, they will take the referral letter but busy talking too much. I had another case where the CHW wrote a referral letter for the child to come immunisation and it was Saturday, the nurse called the patient's and said what is wrong with this child because I can't see anything and it is Saturday today, so I told the CHW to write that mother is working during the week. That is the reaction we get from our colleagues.

Interviewer : Have you presented to the CHW's how the project works?

Interviewee : Yes we did launch this programme to the CHWs and invited them to the launching, all the stake holder and one representative from the clinic. But you know nurses or us, people choose what to do you can't change a person.

Interviewer : How do you think they are resisting?

Interviewee : They are afraid of change and on the other hand we are helping them, the fact is that they say the CHWs are creating a lot of work for us, because the go to the house holds searching for the kids, whereas I expect to see 10 or 15 and they have brought 20 patients over to us, but now at least it's all right.

Interviewer : How do you solve these Problems?

Interviewee : I sit down with the Manager and talk to him/her, if the Manager is not there I talk to the mentors and explained to them what is a Re-engineering and how it works, Even the Ideal clinic when it comes they see that the ideal clinic is inside the Re-engineering. And I explained to them the to have the ideal clinic we must have the Re-engineering, now at least they started to understand us. Otherwise they will just say ''ah that is Mononela and her patients'' and then leave us to it.

Interviewer : How did your OPM help you to resolve this issue?

Interviewee : She sat down with her staff and explained to them that we are not creating more work for them but we are helping them and we are trying to find out those people who are unable to come to the clinic, at least they can go there and find them before it is too late.

Interviewer : How do you understand your role as a team leader?

Interviewee : I just understand that my role is to manage the CHWs and also to supervise them, and to lead them and organise the campaigns. And I also to do supervisory visits with them.

Interviewer : You say you organise campaigns for them, so what I will like to know is how many campaigns have you organised so far with them?

Interviewee : This year we organised only one campaign so far.

Interviewer : What is your role in this campaigns and what is the CHWs role?

Interviewee : When we are doing campaigns I just go with them to the field and we split into groups, if we are 10 or 20 we split into a group of two, two CHWs per house hold, then me too I also go with the other CHWs. Where the other CHWs come across a situation that she cannot solve it herself, I go there myself and assist them.

Interviewer : Do you visit the community?

Interviewee : Yes I visit the community, when the CHWs do door to do I go with them.

Interviewer : What I am trying to find out is, sometimes the team leaders saying the problem is time, they say they can't go to the field because they don't have time because I also have to work here at the facility so I can't go out, so need to find out, how do you manage your time when it comes to working at the clinic and also go and field work?

Interviewee : In our sub- district it is different from the others because here we are permanently allocated to the PHC Re-engineering I am not working in the clinic, I got my own office and they know that I am not permanently

reporting here, all my duties are dedicated to the PHC Re-engineering, I am just here to help so it don't affect my work at all.

Interviewer : Do you interact with other team leaders from other sub-districts, like Giyani maybe?

Interviewee : Yes only when we have meetings, like when we do the PHC Re-engineering reviews, and that is the only time we interact.

Interviewer : What do you do in the PHC Re-engineering reviews meetings?

Interviewee : We usually present the reports for quarterly and if there is any discussion that we must discuss then we discuss them. Also they give some clarity on some information that they have to give us.

Interviewer : In your own view, Why is it you that has to be focused on PHC Re-engineering in this sub-district, and why is Nkomo and Lephepane not only focusing PHC Re-engineering but doing both and you are only focusing on PHC Re-engineering while others have to do both?

Interviewee : When this Project came, our PHC Manager just chose that in my sub-district I want this Project to be worked by sisters who are going to work for me freely and they must have their own offices and they mustn't work in the clinic, but we are still reporting here, but we are not working for both. That is the way she chose it from the beginning it is not our decision.

Interviewer : How do you prefer it to be, to work only for the PHC Re-engineering or to work for the clinic also?

Interviewee : I prefer the way I do it, because it gives me a full time to attend to the PHC Re-engineering, because if I also

worked at the clinic like today there is a shortage of staff, so if it happens that there is a shortage of staff the whole week then it means I won't be able to attend to the PHC Re-engineering's work for the whole week.

Interviewer : Do you feel that your work loud is Manageable?

Interviewee : Yes it is Manageable.

Interviewer : What are the challenges you are facing as a team leader?

Interviewee : We don't have enough stationery, like when we are tracing the patient we got the tool that we must use, so in my office because I am not working alone I am working with the clinic staff and we don't have a copying machine, I don't have access or any way to make copies, so I have to rely onto the NGOs and sometimes the NGOs will say they don't have ink or they are still busy, so it means there will be no tracing tool for that month or that week. The other challenge is means of communication because mostly if I want to communicate with my CHWs I have to use my own airtime. And number three is we don't have computers, we are still using manual, so if I have to send statistics I have to come here or go to Kgapane sub-district and I have to use my own money for transport, because we don't have our own transport. Usually we are using the lab transport but now they don't take anything from the clinics, only blood or the things they came to collect. The last one is the transport issue, because if I have to do a supervisory visit far away from here, I have to use a transport and our office don't have transport, so sometimes I get stuck in one place.

Interviewer : You spoke about a tool, what do you mean by that?

Interviewee : There is a tracing tool that the CHWs and the team leaders use to report about the patient's condition and we also have to write the outcome on that tool. So if we don't have that tracing tool the CHWs will just come and report verbally. And if it is verbally I won't have any evidence of what the CHW is telling me and if they really did go and do the patient tracing and I won't know for sure if the patient died or is still alive.

Interviewer : So it means here you just have predict what really happened between the CHW and the patient?

Interviewee : Yes

Interviewer : So the Government don't supply you with the tracing tools?

Interviewee : They do but it is not enough, only less than 10 per quarter not per month, and usually they print more than 20 per month or per week, and each CHW must have her own.

Interviewer : What can be done to improve the service?

Interviewee : If they can give us more stationery and also for the transport if the local area or per two clinics they give us one vehicle, like me and Phakhula the Team leader from Matswhi or Kgapane we can use one Vehicle because it is in the same line. The other day we can go to that side of Kgapane and then the next we can go this side in Senopela, I think that could be better. And for equipment I think maybe Anova can assist with the computers.

Interviewer : In closure I think we have asked you everything but there is only one part that I want to understand clearly, the issue of the CHWs, Do they often report here or where do they report to, how is the structure?

Interviewee : Because they stay far away I communicated with their NGOs that the way they use to report before they came to the Re-engineering they must continue reporting like that, so they are still reporting to the NGO and Clinics where they are staying, they are the ones for control the goods. But if they not coming to work I told them that they must inform me and also their Manager, because sometimes they say they reported to their manager and the manager said they must not come to work, but then the Manager will say that the CHW said she spoke to me, so we realised that they were playing with our minds. Because the NGOs are the ones who are paying them I said they must report to them.

Interviewer : Thank you for your time and your views, we highly appreciate that, I think we asked you almost everything, or is there anything else you will like to Add?

Interviewee : No, Thank you.
