# Supplementary material for: Qualitative assessment of facilitators and barriers to HIV programme implementation by community health workers in Mopani district, South Africa
Source: PLoS One. 2018 Aug 30;13(8):e0203081. doi: 10.1371/journal.pone.0203081 (PMC6117027; doi:10.1371/journal.pone.0203081)
Supplement: S5 Information — (PDF) [file pone.0203081.s005.pdf]

Date conducted: 14/06/2016 Interview type: FGD

Date transcribed: 17/07/2016 Site: Facility 1

Interviewer: NZ

Interviewer : Is there anything that you find very rewarding or interesting about doing household visits?

Interviewee 1 : While we are working in our community, especially since the program of giving us equipment's, such as blood pressures and scales we see a lot of improvement in how we work. When people see us pass by they call us to come and test BPs and when we test we are able to refer them to the clinic. Some people had high blood pressure that is higher than normal and they were lazy to go the clinic. Once we visit them and check their pressure we are able to refer them to the clinic.

Interviewee 2 : Thank you for giving me time, it is important for and we getting helped with our community, at home they didn't understand the reason when we tell them to go test or to weighed. They used to tell themselves that the sickness is just for a while it's going to pass. Since we have received this equipment's people see the importance of being referred to the clinic, because they are first texted at home and they were able to see the unseen. It is important and it is very helpful for you giving us the scales and the BP machines our community has benefited. In the beginning when we told them to go to the clinic without texting them they didn't take it important, now since they first get tested at home they can see that their BP is not normal they are able to understand the importance of being referred to the clinic.

Interviewee 3 : People didn't see the need to go to the clinic to check their blood pressures. It is important for everyone and very helpful to the community. Now people see the importance of being referred to the clinic.

Interviewee 3 : Thank you. While working in our community, the community likes us. When they see us they welcome us. The equipment's that we have received are appreciated by the community when we visit them and by doing the tests and they liked being checked or tested.

Interviewee 4 : What excites me is visiting pregnant people especially young people. Those kids get excited when they see me because they get pregnant at a very young age. When I visit I encourage them to go to the clinic and I also write a referral. They do a follow up and also book on time. They also follow up on the dates have to go back to the clinic and how are they supposed to live and the dates that they will go to labour while helping them. They welcome our help at all time.

Interviewee 5 : I am very excited that we received equipment's that allow us to test blood pressure people at home they seem like they like it when we come with them. We refer them to the clinic and they come back to us with feedback and we also go check if they really went.

Interviewer : Is there anything that you find very difficult or challenging about doing household visits?

Interviewee 1 : One of challenges that I experience, sometimes when I visit a certain household and tell them that I teach about the health illnesses that exist and then they tell me that

they have a sick person in the house, what I usually do I write a referral letter to the clinic and you find that the person is unable to go to the clinic, the reason is that the person is unable to walk .This is a challenge that I have experienced.

Interviewee 2 : Another challenge you find that a person is on treatment but does not have food to eat. Some do not earn grant or they don’t even have a child to earn the grant on their behalf. it is not easy for us to let the person take the treatment. When I get to the persons house you find that he does not have food. The treatment that the person takes requires eating before taking the pills and the person does not have food to eat to be able to take pills.

Interviewee 3 : They treat us well. In our community they don’t tell us to turn back when they see us coming, they are very welcoming. When we pass by these houses some invite us to visit them. Our relationship is good they love us and we also love them.

Interviewee 4 : The people in the community treat us well. You find that during weekends they come to our house to tell us their problems. The community trust us. Sometimes when they see us they are able to tell me that yesterday I didn’t see you I wanted something from the clinic. They understand that they should work with certain people.

Interviewee 5 : Sometimes we find out that a person is staying alone we are able to clean for him/her and we even make soft porridge for the person to eat. Some we are able to wash their clothes so that they can be clean.

Interviewee 6 : We are able to go to the clinic to fetch their treatment only if they are not defaulters. We always give these

patients enough information so that they are able to go to the clinic to get their treatment when we not around.

Interviewee 7 : When we not working the community is able to come and see us to share their problems and we solve them. We don’t consider if it’s a weekend or not.

Interviewer : Do you think people listen to you when you discuss HIV and other health issues with them?

Interviewee 1 : They listen to us. When we have referred them to the clinic they come with their referral letters and this shows that they listen to us

Interviewee 2 : They listen to us when we tell them to come to the clinic to get tested.

Interviewee 2 : They listen to us. When we look back, there were number of people who were sick in the community, since we have started working the number has decreased. Now people are taking their treatment very well.

Interviewee 3 : They listen to us, we also tell them what might happen if they don’t take their treatment or when they not consistent. When we teach them they are able to be consistent.

Interviewer : How much training did you receive before you had to visit households?

Interviewee 1 : We were trained a lot about TB. This allows us to teach people that we visit about TB precautions, symptoms and how it can be cured.

Interviewee 2 : When we started with this job we first went to workshop and training. When I remember correctly they

trained us for 3 weeks about different illnesses and some would occur once or twice a week, even now we are still trained. If there is something that we do not understand we go to the clinic to ask them to train us. They help us a lot. If we forget we go back and ask them to train us. In all trainings that the nurses get on illness we are also trained.

Interviewee 3	: When we teach people about TB. We teach them that when they cough more than one day they must go to the clinic to go consult. These days when a person start coughing, he or she must go to the clinic to consult so that they can get treated.
Interviewee 4	: A person must know that when they go to the clinic they should have privacy between the person and the nurse.
Interviewer	: What kind of training did you get?
Interviewee 1	: When we started we got a home-based training certificate that covers all the illnesses that we will be working with at these households. Besides that the government is able to ask us what kind of training we might like to have so that they can provide. Like now some of the CHWs, are at a certain village where they are being trained.
Interviewee 2	: There are workshops that the government provide. When we want to be trained on TB and HIV we ask them to train us.
Interviewee 3	: We are trained on nutrition

Interviewee 4 : Mental health

Interviewee 5 : Breast feeding

Interviewer : How well did the training prepare you for visiting households in the community?

Interviewee 1 : They prepared us well; the training that we have received from the reengineering has increased our knowledge and taught us more about HIV and TB. This will enable us to save lives of people in the community. These trainings truly help us because in our community we rarely find people who are bedridden. Bedridden are only people who come back from Johannesburg. Here in the community how does one become a bedridden when there is a clinic?

Interviewee 2 : All the training has help us because when we refer people to the clinic they are able to go and when we are talking to them it shows that they understand what we are talking about.

Interviewer : What should be added to the training to make it better?

Interviewee 1 : As time is changing we want them to train us and we welcome that. We want to be trained so that we can improve. Things are changing; just like TB when we receive new training we can see that there is change compare to previous training. All this help us and the community as well.

Interviewee 2 : When we look back on HIV when it all started people were receiving treatment when their CD4 count was 300 and now that has changed people are now receiving

treatment when their CD4 count is 500 or when they are sick. Long time ago the reason we had bedridden is because people had to wait to reach 350, even though they were sick they couldn't go to the clinic. Now it depends on their condition to receive treatment.

Interviewer : What other training do you think CHWs need?

Interviewee 1 : We need diabetic training.

Interviewee 2 : I also second the diabetic training because since we have been given medical equipment's to work with; when we get to households they ask if we have diabetic tests. I think we should get the diabetic test. People want to be helped a lot.

Interviewee 3 : If possible to get pregnancy test that we can use for teenagers after we have had a small talk with them. Another thing would be malaria equipment's. When we get to these houses they ask us if we have such equipment's.

Interviewee 4 : If we can be trained on Pap smear. People have been encouraged to do Pap smear and they are even scared. This was going to help the community.

Interviewer : What is your workload like?

Interviewee 1 : It's good because we know the number of houses that we should visit by depending on what the program says.

Interviewee 2 : On the number of houses that we should visit you find that when we visit a certain household a patient does not

want you to leave and every household has its own time allocated this makes us to make the program fail because we end up not reaching the target households; 9 households a day.

Interviewee 3 : In situations that we have to cook for a person in order to take treatment, it can make us not to reach the targeted number of households that we need to visit for that day. Sometimes we try to sacrifice but we fail.

Interviewee 4 : Our work is good. Sometimes we end up not reaching targeted number of houses to visit because you find that I spend two hours with a patient.

Interviewer : What do you do when you spend two hours with a patient?

Interviewee 4 : In cases where person stay alone, when I get to these houses I first clean up and make food for the person to eat. After doing that I first have to wait to see if this person is taking his or her treatment while creating a conversation.

Interviewee 1 : To add on that, after making food, the person will say I don't have water. We will go fetch water and when we come back the person will say I don't have food and there we will have to cook. This one of the reason we spend so much time with them.

Interviewer : What should change to help you reach more people in the community?

Interviewee 1: The government must provide more soft porridge for people so that when we visit households which a person

stays alone we could just prepare the instant porridge for them so that they take the required treatment. This will make it easier to move on to the next house. The program of giving sick people food must continue. All this it will make things easier.

Interviewee 2 : To add on that they should give them liquids to make fire. This will save us time on setting fire to prepare one.

Interviewee 3 : When the social worker gives food to the community he/she must make sure that that every disadvantaged households get food.

Interviewee 4 : If the government would listen to our request we think we would reach our target and we were not going to be delayed. We are requesting them to take these ideas serious. We don't know who is fit to receive food. I think they should give it to everyone so we can be able to reach more houses. When we leave the clinic we would leave with this food to give the people that are in need.

Interviewee 5 : How would it be if they decrease the number of people that we need to see in a day from 9 to 6 households?

Interviewer : Do you experience any challenges follow up on patients who do not visit the clinic to collect their medication?

Interviewee 1 : Some patients when they have to come fetch their treatment at the clinic they have to travel for a long distance from their house to the clinic. While walking they have to rest on the way because they get tired. This is difficult for them.

Interviewer : Is it easy or difficult to get a list of defaulters?

Interviewee 1 : It's easy. When we visit the clinic they are able to tell us the number of people who are not taking treatment and they tell us to do follow up's on those people. We follow up on them to come fetch their treatment

Interviewee 2 : Another thing that we find it difficult is patient's information because some write incorrect information and when we do follow up we end not finding the person because of the incorrect information. If the patients wrote the correct information we are able to trace and follow up.

Interviewee 3 : It is easy for me, because I like writing referrals when they get to the clinic they get help and the patients end up knowing what is making them sick.

Interviewer : What do you find is the best way to contact patients?

Interviewee 1 : Is when the nurses have given us the patient's personal details where we end up knowing the physical address, we are able to go fetch the person and come back with her/him to the clinic.

Interviewee 2 : When we have received the person's information from the clinic. When we have the physical address we are able to go to that person's place and when we get there we are able to write a referral so that the person comes with it to the clinic. The next day we do a follow up if that person has visited the clinic. If they didn't go we would want to understand the reason.

Interviewee 3 : Do you experience any challenges following upon TB patients?

Interviewee 4 : Some TB patients want to DOT (directly observed treatment) themselves they don't want us to assist them or help them, and this people are mostly the ones who are likely to be defaulters.

Interviewee 5 : Sometimes when we see that we might find a challenge we invite the nurses to accompany us so that we can talk to the person so that they will end up following up on their treatment.

Interviewee 6 : Some take treatment when we test their spectrum it says positive because they still cheat by smoking and we see prove by finding cigarette left overs at the persons house and you find that he is the only one smoking in the house. It ends up looking like he is not getting the right treatment from the clinic.

Interviewer : Do you experience any challenges following up on pregnant women?

Interviewee 1 : Some people who are pregnant they don't want to come to the clinic to book. Now it is winter people wear jerseys and you hardly notice if someone is pregnant when you teaching. When is seven months that's when they realise that it is too late they have to go book and when they go they even wear tight dresses that shows that they are pregnant. The nurses will ask us why we did not refer the person in time but we failed to see that she is pregnant when we were visiting her. When she decides to go to the clinic you find that it is too late.

Interviewee 2 : Young adults are difficult; you find that sometimes when we do follow up they fail to visit the clinic while we wrote a referral letter. They fail us and the nurses. When they are told to come they say that they will come and they end up not coming faithfully and time is going

	even though they are told about the consequences. We find it difficult we end up not knowing what to do.
Interviewee 3	: To add on young adults, we see them pregnant but after a while they are no longer pregnant and you not sure what happened. Situation like these give us problems.
Interviewer	: How do you work with the social workers in your ward? What do you think about the social workers in your ward?
Interviewee 2	: When we find challenges on households that we visit such as lack of food and social grants we interact with the social workers so that they are able to help these people.
Interviewee 1	: On children without parents we are able to talk to them and write a referral letter to a social workers so that they can be help them because they are orphans. We also write a referral letter to people who are mentally ill to go see social workers because you find that they are a problem at home, so that they can see how to help them.
Interviewee 3	: When the social workers don't know someone's physical address they call us to assist them to find that person's physical address. If there is a problem we come back with the person.
Interviewee 4	: What we like about our social workers is that they work with us well and we have a mutual relationship if they need help they tell us and if we want help we tell them. What I like about them they know how to be confidential if we tell them that they must not tell anyone that they heard something from us they keep the

secret and assist that person who might be in need. We are one.

Interviewer : What do you think is the role of social workers?

Interviewee 3 : Social worker assists people who are in need. When we go to households we are able to identify the people who might be in need the social workers assist those people.

Interviewee 2 : On children that are orphans who do not have IDs the social workers are able to help the children to register for IDs and social grants including those who do not have birth certificate. They get proper help.

Interviewer : Do you encounter challenges with social workers?

Interviewee 1 : We have never had any challenges they try by all means to follow up exactly what we have told them. Especially to those who are poor, they are helped.

Interviewer : What do you think of community leaders?

Interviewee 4 : Our community leaders are people who understand. When there is a problem in the community we first take the problem to these leaders before it can go to the police station. We see their importance and role.

Interviewee 2 : They treat us well. When we ask something they give us. At the end of every year we have a campaign where we report back to the community on what we have done throughout the year, we invite the community leaders to come and they have never disappointed us and not

	come. The leaders even ask us if we might want helped from the report that we gave.
Interviewee 3	: When there is a community meeting the community leaders are able to tell the community to go to the clinic to test their spectrum for TB. They are not scared to tell the community.
Interviewee 5	: When there is community meeting they are able to invite us to come and address the community.
Interviewee 1	: When there is a community they are able to tell the community to work with us even if we are not present at the meeting. They tell them to call us if we are working in another area because we are going to help them.
Interviewee 3	: Even the King of this area reminds them that they should not lock people who are sick in the house they must get help that we the CHWs offer to the community.
Interviewee 4	: When we not around the community leaders even give announcements on our behalf. We work well with each other
Interviewee 5	: Our king which works with community leaders also reminds the community that when they sick they must go to the clinic they must not lock people in the house. They are told to utilise the CHWS and also go to the clinic to get help.
Interviewer	: How do you deal with negative influences of community leaders?
Interviewees	: We have never experienced that (all responded)

Interviewer : What do you think of traditional healers?

Interviewee 2 : We work with them well. When someone goes to the traditional healer to consult the healers first ask them if they had consulted the clinic. When they see us visiting households they ask hand gloves from us. So that when people visit them they can treat them wearing hand gloves.

Interviewee 3 : We work well with the traditional leaders when they see that someone's health is not improving, they call us to come take the person to the clinic. When they have a patient they are able to call the clinic to come see the patient, they allow us to work with them and we are able to go visit the patient that they are treating.

Interviewee 4 : The traditional healers they are able to say that what we are told in our meeting is that we cannot heal TB, TB is only cured at the clinic. When they see a patient that might have TB they are able to refer that person to the clinic.

Interviewer : How do you deal with negative influences of traditional healers?

Interviewees : We have never had such (all replied)

Interviewer : Do you have anything to add on what we have discussed?

Interviewee 1 : Since the reengineering program is led by ANOVA we can say that ''hala-hala" (show of appreciation)

Interviewer	: Thank you
Interviewees	: You welcome!
