# Supplementary material for: Qualitative assessment of facilitators and barriers to HIV programme implementation by community health workers in Mopani district, South Africa
Source: PLoS One. 2018 Aug 30;13(8):e0203081. doi: 10.1371/journal.pone.0203081 (PMC6117027; doi:10.1371/journal.pone.0203081)
Supplement: S6 Information — (PDF) [file pone.0203081.s006.pdf]

Date conducted: 25/05/2016 Interviewee type: FGD

Date transcribed: 27/06/2016 Site: Facility 3

Interviewer: NZ

Interviewer : My first question is what is your experience of the CHW's?

Interviewee 1 : My experience is that they are working, they are part of us and they are reducing our patient burden

Interviewer : Any other sister who wants to share her experiences with the CHW's?

[Quietness]

Do you communicate or interact with them? We want to know any experiences you have of them

Interviewee 2 : They make our job easier because they are doing house to house visits so they are able to reach the people which the clinic is unable to reach.

Interviewee 3 : They make our job easier because we are able to send them to search for defaulters and indeed they will find those patients and sometimes they come with them. We work with them very well and when we ask them to do something for us they do it

Interviewee 4 : We give them the names of the defaulters and they will go look for them and bring them back. For instance, if there is a child who didn't get immunization they will bring the child to the clinic for immunization

Interviewer : Ok let me pick up from the point of defaulters, what you think are the main reasons people default on treatment in this area?

Interviewee 1 : In most cases is that when they feel that they are better they stop taking treatment. Sometimes it's because of many tables they are supposed to take, it discourages them and they stop.

Interviewer	: Any other views?
Interviewee 3	: Some of them it's because they don't want to disclose their conditions. So when they come to the clinic and meet other people it discourages them because they don't want people to know that they are ill.
Interviewer	: What is the cause of that?
Interviewee 3	: It's because of stigma, if the person is positive they feel like they are not welcome and wanted. So it's the stigma causing this
Interviewee 1	: Others are defaulting because they are using traditional medicine/Muthi. So they will stop taking pills and they will use muthi.
Interviewer	: How do you deal with these challenges?
Interviewee 2	: When our patient has defaulted we use the CHW's
Interviewer	: How do you use them?
Interviewee 2	: We inform them and ask them to trace them
Interviewee 1	: In some instance you find that they go to visit the patients and some of them will not be happy that we send the CHW to their houses they don't want people to know. They will come and tell us that they are not happy with us sending the CHW.
Interviewee 2	: Sometimes we use our cell phones to contact them because we have their phone numbers and when they come to the clinic we explain to them why we are phoning them
Interviewer	: Ok let me build more from this and ask how do you trace the defaulters?
Interviewee 1	: As they have stated that we send the CHW's; we give the CHW's a list of people we want them to trace. We give them a list with names of defaulters and they go out looking for them and the patient will come back

Interviewer	: Is a defaulter list being generated?
Interviewee 3	: Do we have it
Interviewee 1	: Yes we have, for these people to come back we use the list
Interviewer	: Who is the person responsible for generating the list?
Interviewee 2	: Data capture
Interviewee 1	: He is able to see on his system who defaulted and he gives us a list
Interviewer	: How can the CHW's improve on dealing with the CHW's?
Interviewee 4	: I think they can improve by getting further training
Interviewer	: They must be trained
Interviewee 3	: If they are trained they will know that person's information is confidential and trained on how to interact with people. Trainings will help them improve and also get more knowledge
Interviewer	: Do you all agree or there is someone with a different view?
Interviewees	: Agree (all agreeing)
Interviewer	: In a situation where someone is defaulting how do you deal with it as facility nurses?
Interviewee 1	: When the CHW bring them to the clinic we take them to the lay counsellors so they can talk to them and they will be able to find out what made them to default and then after they are able to take their treatment again
Interviewer	: What can be done to improve the process of defaulter tracing?
Interviewee 2	: I think what is needed is a phone and we would be able to get them as soon as possible if we had a phone. The phone is the simplest way of tracing them, but now we don't have a phone and the one we had before it was not dialling out and we couldn't phone our patients

Interviewee 1 : It was only for the department to call when they need something

Interviewee 4 : Yes when the patients get a phone call from the clinic they come running, they respond very fast. Usually we use our personal cell phones to call the patients because we don't have a facility phone. When we call them we are able to get them, so as the sister have stated we really need a phone

Interviewee 3 : Another thing is that the CHW's need to be supported, for example if there is a patient who needs to be visited, if sister Chabalala [team leader] visits the household with them it also motivates them

Interviewer : Ok you are saying that the CHW's should be supported by the facility nurses?

Interviewee 3 : Yes they need support from the nurses

Interviewee 1 : And also include the NGO's

Interviewer : Ok the NGO's needs to be included, how can the NGO's be included?

Interviewee 1 : For example, Anova has cars, if the patients stay far like in Nkomo A or Randanhloko at the beginning of the village, you can take one of the CHW or take one nurse to trace the patients

Interviewer : So what you are saying here is that the basic challenge is transport?

Interviewee 1 : Yes we don't have transport

Interviewer : Is there anyone who wants to add?

(Quietness)

Ok let's go on, what do you think helps the defaulters to return back to the clinic?

Interviewee 1	: I think it's the approach, when they visit them at home they must approach them well and when they are here at the clinic we must approach them well
	(Door opening and one sister being called out)
Interviewer	: Ok let us proceed, what do you think are the causes for people to default on treatment?
Interviewee 4	: When their illness get worse they return for treatment. When they feel that the illness is unbearable they return for treatment
Interviewer	: Do you want to add something?
Interviewee	: No
Interviewer	: Ok let me follow up on my previous question, what makes other people not to come back to the clinic?
Interviewee 1	: Maybe is the approach they got on the first day
	(Movement in and out; the other one comes back and the other one go)
Interviewer	: Ok we are almost done; we are only left with few questions. Let me go back to my first set of question and we are done. How often do the CHW's visit the clinic?
Interviewee 1	: Every day except on weekends
Interviewee 2	: They do work on some weekends if they have appointments with their patients
Interviewer	: Do you have meets with them?
Interviewees	: Yes (all agreeing)
Interviewer	: What do you discuss in those meetings?
	(Quietness)
Interviewee 1	: We tell them how to communicate with the people in the houses. Sometimes they teach the people and then they tell

them to visit the clinic. When they come to the clinic they will tell us that yesterday the CHW came to my house and was teaching me about health issues and that made me to come to the clinic to know my status.

Interviewee 2 : Sometimes they discuss the stumbling blocks of their program

Interviewer : Can you please share with us what are those stumbling blocks?

Interviewee 2 : Some families don't allow them access

Interviewer : Why don't they allow them access?

Interviewee 2 : They say it will draw people's attention because that will mean there is someone in their family who is HIV positive

Interviewee 1 : For example, if someone in the house has TB and you refer the CHW they won't have a problem, but if there is a case of HIV they won't allow them

Interviewer : Why is that the case?

Interviewee : They don't want people to know (all agreeing)

Interviewer : Ok let move on, how do the CHW's affect your workload?

Interviewee 2 : They make our job very easy because if there is someone sick we don't see those patients since they are the ones who visit these patients at their households. They Dot the patients and this help us as nurses because we don't have to Dot because the CHW's are able to do this. They agree with the patient on the time of taking treatment and they will visit them.

Interviewee 1 : In most cases they give people health education as we know that prevention is better than cure. This means we won't have a lot of people who will come to the facility with the same problem which the CHW's are teaching them about

Interviewer : Do they refer these clients to the clinic?

Interviewees : Yes they refer them (agreeing)

Interviewer	: How is this referral system working so far? How does it work or what are your views about this referral system?
Interviewee 1	: It is good
Interviewer	: Is it helpful or you finding it not useful?
Interviewee 2	: It is helping us because if the CHW's were not there it was supposed to be us who trace the patients. They are reducing our workload
Interviewer	: Be for I close, how do you think we can improve the work of the CHW's?
Interviewee 1	: Trainings or workshops
Interviewee 2	: Increasing their numbers will help because here at Nkomo B the village is too big but we have few of them. Actually you find that a CHW has a patient who stays far away and it is also difficult for her to reach that patient
Interviewee 3	: They also need equipment's
Interviewer	: What type of equipment?
Interviewee 3	: They need BP machines and temperatures because you find that they visit a household they will be able to pick up a lot of things earlier
Interviewee 1	: For example, if they weigh a child and they find that the child is underweight they are able to refer that child to the clinic and you will find that the mother was not bringing back the child for check-ups.
Interviewee 2	: They also need protective clothes, and you find that it's raining and they don't have umbrellas, and sometimes it's very hot and in this area it's too hot.
Interviewer	: Ok this side they don't have umbrellas?
Interviewee 2	: Do they have umbrellas? (Asking her colleagues)

Interviewee 3	: Few of them have umbrellas
Interviewee 1	: Only Re-engineering people have the umbrellas but not the other home base carers. They also need to be supplied with T-shirts like those ones given to Re-engineering people. They have T-shirts with health information on it and this makes the patient to have confidents that this person is from the department and they can see the emblem of the department. This means they are the nurses, but if they don't have attire people will doubt them. They need those T-shirt like the one for Re-engineering
Interviewer	: Now it's only the CHW's who have the T-shirts while home based carers don't have anything
Interviewees	: Yes (all agreeing)
Interviewee 2	: Another thing is the issue of stipend; they need the stipend; even a small amount so to encourage them
Interviewer	: How much would you say is small amount?
Interviewee 1	: At least it must be above R3500, it would be better
Interviewee 2	: Yes, because every day they are here with us and they knock off after half past four
Interviewee 3	: Yes, these people are working and they are staying very far and they are coming here everyday
Interviewer	: You all agree on R3500 and above
Interviewee	: Yes (agreeing)
Interviewee 1	: The other thing is that it would be better if some of them who can be trained to be nurse they must be trained so that it can also motivate the young ones who are at homes and not doing anything. This will encourage them to become community health care workers

Interviewer	: Ok is there anything else that you want to discuss or you think I didn’t ask? Anything I didn’t ask or we didn’t touch<br><br>(Quietness)<br><br>There is nothing? If there is nothing I would like to thank you for your time and views, highly appreciated, thank you.
