# Supplementary material for: Qualitative assessment of facilitators and barriers to HIV programme implementation by community health workers in Mopani district, South Africa
Source: PLoS One. 2018 Aug 30;13(8):e0203081. doi: 10.1371/journal.pone.0203081 (PMC6117027; doi:10.1371/journal.pone.0203081)
Supplement: S10 Information — (PDF) [file pone.0203081.s010.pdf]

EVALUATION OF WARD-BASED OUTREACH TEAMS: FOCUS GROUP DISCUSSION – FACILITY NURSES

Evaluation of Ward-Based Outreach Teams

Anova Health Institute: Consent form and information sheet

INTRODUCTION

Hi, my name is________________________________ I am working for the Anova Health Institute. I would like to invite you to participate in an evaluation that we are conducting. The study is called "Evaluation of Ward-Based Outreach Teams." Before you decide whether or not to participate, I would like to tell you about the study and answer any questions that you have. If you agree to participate, you will be asked to sign a consent form. You will be given a copy of the information sheet to keep. Please note that your participation is voluntary, and you may choose to withdraw from the study at any time. There will be no negative consequences if you choose not to participate, and your choice will not affect any of the health services that you access.

Why are we doing the study?

We are interested in understanding how the community health workers operate in the community and whether or not their efforts are helping people access medical care. It is important for us to collect this information in order for us to address any challenges and improve systems that will in turn improve the community's access to medical care. If you agree to participate in this evaluation, you will participate in a focus group discussion with other facility nurses. A focus group discussion is a method of qualitative data collection from participants with similar backgrounds and experiences. The focus group discussion will be guided by a facilitator who will introduce the topics for discussion and assist the group to participate in a natural discussion. Participants are allowed to agree or disagree with each other. Please feel free to discuss any issues that you feel are important for you.

What will happen during the study?

If you agree to participate, we will interview you with a standard questionnaire. The questionnaire includes questions about your experiences with community health workers and accessing medical care at your local clinic.

What will happen if I agree to participate in the study?

You will be given time to consider participation and all your questions will be answered before you make your decision. If you agree to participate in the evaluation, you will be asked to sign a consent form. If you sign the consent form this will mean that you have read this document, that the evaluation has been explained to you, that all your questions were answered and that you agree to participate in the evaluation. If you agree to participate in the evaluation, the discussion will be audio recorded. You may withdraw consent from participating in the evaluation at any time and this

will not affect your access to medical care. In that case, all information collected will be destroyed with no negative consequences to you.

Risks

We do not think that there is any risk to you from completing the survey. We will treat your answers with confidentiality but because this is a group discussion we cannot guarantee that other participants will not share what you have said. We will not share or discuss your answers with community health workers, and we ask that you also do not share anything that other participants say in this group with anybody else.

Benefit

There is no direct personal benefit to you from participating in this evaluation. However, findings of this evaluation will be used to optimize the CHW system and this may benefit you and the community in future.

Confidentiality

All the information that you provide to us will be kept confidential. We will not mention your name in any of the reports from this evaluation.

If you need more information or you have any questions regarding the evaluation, please feel free to contact

Prof. Remco Peters at 011 581 5011 or Dr. Jean Railton at 015 307 4893.

or

Human Research Ethics Committee
Prof. Cleaton-Jones
Administrator and Chair
University of the Witwatersrand
Wits Research Office
10th Floor Senate House
East Campus
Johannesburg
Tel: 011 717 1234
Fax: 011 717 1265

 Study ID: F ___/___/___/___/___

Do you understand what you have read/has been read to you? Please cross an answer below?

☐ Yes ☐ No

Do you agree to participate in this study? Please cross an answer below?

☐ Yes ☐ No

_________________________ _________________________________

Participant Name Date

_________________________ _________________________________

Participant Signature Date

Please obtain a signature from an adult witness if the participant is unable to read and write.

_________________________ _________________________________

Witness Name Date

_________________________ _________________________________

Witness Signature Date

_________________________ _________________________________

Interviewer Name Interviewer Surname

_________________________ Date _________-_________-20_________

Interviewer Signature

Do you understand what you have read/has been read to you? Please cross an answer below?

☐ Yes ☐ No

Do you agree to participate in this study? Please cross an answer below?

☐ Yes ☐ No

_________________________ _________________________________

Participant Name Date

_________________________ _________________________________

Participant Signature Date

Please obtain a signature from an adult witness if the participant is unable to read and write.

_________________________ _________________________________

Witness Name Date

_________________________ _________________________________

Witness Signature Date

_________________________ _________________________________

Interviewer Name Interviewer Surname

_________________________ Date _________-_________-20_________

Interviewer Signature

EVALUATION OF WARD-BASED OUTREACH TEAMS

Formal Signed Consent Form for Audio Recording

The researchers have my permission to audio record this interview/ focus group discussion (circle as appropriate) in order to include all important information. This has been explained to me in the participant information sheet. The evaluation has been explained to me and all my questions were answered to my satisfaction.

________________________ ________________________________

Participant Name Date

________________________ ________________________________

Participant Signature Date

Please obtain a signature from an adult witness if the participant is unable to read and write.

________________________ ________________________________

Witness Name Date

________________________ ________________________________

Witness Signature Date

________________________ ________________________________

Interviewer Name Interviewer Surname

________________________ Date _________-_________-20_________

Interviewer Signature

EVALUATION OF WARD-BASED OUTREACH TEAMS

Formal Signed Consent Form for Audio Recording

The researchers have my permission to audio record this interview/ focus group discussion (circle as appropriate) in order to include all important information. This has been explained to me in the participant information sheet. The evaluation has been explained to me and all my questions were answered to my satisfaction.

_________________________ _________________________________

Participant Name Date

_________________________ _________________________________

Participant Signature Date

Please obtain a signature from an adult witness if the participant is unable to read and write.

_________________________ _________________________________

Witness Name Date

_________________________ _________________________________

Witness Signature Date

_________________________ _________________________________

Interviewer Name Interviewer Surname

_________________________ Date _________-_________-20_________

Interviewer Signature

LAY GROUND RULES

The facilitator, with the participation of the group, will lay ground rules for the discussion

- Turn off cell phones
- Respect for the views of others
- Respect others' confidentiality – meaning not to reveal what others have said here today
- Try not to interrupt each other—give everyone a chance to speak their mind, and respect opinions that are different from your own.
- Everyone should feel free to participate as much as they would like.
- You should feel free to use the language you use with each other in normal, everyday discussion. However, please refrain from language that is insulting or offensive to one another.
- Do you have any other suggestions for ground rules?

Interviewer Script: I will now ask you a few questions about your experience with community health workers

▪ **What has been your experience of the CHWs?**<br>▪ [probe]: How often do CHWs visit the clinic?<br>▪ [probe]: How often do you have meetings with them?<br>▪ [probe]: If so, what do you discuss in the meetings?

▪ **How do CHWs affect your workload at the clinics?**<br>▪ [probe]: How does this affect patient linkage to care?<br>▪ [probe]: How does this affect the quality of data collected?<br>▪ [probe]: Has workload challenges (if this is the case) been addressed with the facility managers?<br>▪ [probe]: If so, have there been any improvements since?

Interviewer Script: I will now ask you a few questions about defaulting patients

▪ **What do you think are the main reasons people default on treatment in this area?**<br>▪ **How does the facility deal with defaulters?**<br>▪ [probe]: Is a defaulter list generated?<br>▪ [probe]: What is the role of the CHWs in this process?<br>▪ [probe]: How can CHWs improve in dealing with defaulters?<br>▪ [probe]: If defaulters are not dealt with, why is this?<br>▪ [probe]: What is needed to optimize defaulter tracing?<br>▪ [probe]: How can we optimize defaulter tracing?

▪ **What do you think helps defaulters return to the clinic for treatment?**<br>▪ [probe]: Why do you think some defaulters don't return to the clinic?

Interviewer Script: Thank you very much for your time and opinions.
