# Supplementary material for: Qualitative assessment of facilitators and barriers to HIV programme implementation by community health workers in Mopani district, South Africa
Source: PLoS One. 2018 Aug 30;13(8):e0203081. doi: 10.1371/journal.pone.0203081 (PMC6117027; doi:10.1371/journal.pone.0203081)
Supplement: S11 Information — (PDF) [file pone.0203081.s011.pdf]

EVALUATION OF WARD-BASED OUTREACH TEAMS: IN-DEPTH INTERVIEW – SOCIAL WORKERS

Evaluation of Ward-Based Outreach Teams

Anova Health Institute: Consent form and information sheet

INTRODUCTION

Hi, my name is________________________________ I am working for the Anova Health Institute. I would like to invite you to participate in an evaluation that we are conducting. The study is called "Evaluation of Ward-Based Outreach Teams." Before you decide whether or not to participate, I would like to tell you about the study and answer any questions that you have. If you agree to participate, you will be asked to sign a consent form. You will be given a copy of the information sheet to keep. Please note that your participation is voluntary, and you may choose to withdraw from the study at any time. There will be no negative consequences if you choose not to participate, and your choice will not affect any of the health services that you access.

Why are we doing the study?

We are interested in understanding how the community health workers operate in the community and whether or not their efforts are helping people access medical care. It is important for us to collect this information in order for us to address any challenges and improve systems that will in turn improve the community's access to medical care. If you agree to participate in this evaluation, we will do an interview with you. Please feel free to discuss any issues that you feel are important for you.

What will happen during the study?

If you agree to participate, we will interview you with a standard questionnaire. The questionnaire includes questions about your experiences with community health workers and accessing medical care at your local clinic.

What will happen if I agree to participate in the study?

You will be given time to consider participation and all your questions will be answered before you make your decision. If you agree to participate in the evaluation, you will be asked to sign a consent form. If you sign the consent form this will mean that you have read this document, that the evaluation has been explained to you, that all your questions were answered and that you agree to participate in the evaluation. . If you agree to participate in the evaluation the interview will be audio recorded. You may withdraw consent from participating in the evaluation at any time and this will not affect your access to medical care. In that case, all information collected will be destroyed with no negative consequences to you.

Risks

We do not think that there is any risk to you from completing the survey. We will treat your answers with confidentiality. We will not share or discuss your answers with the community health worker.

Benefit

There is no direct personal benefit to you from participating in this evaluation. However, findings of this evaluation will be used to optimize the CHW system and this may benefit you and the community in future.

Confidentiality

All the information that you provide to us will be kept confidential. We will not mention your name in any of the reports from this evaluation.

If you need more information or you have any questions regarding the evaluation, please feel free to contact

Prof. Remco Peters at 011 581 5011 or Dr. Jean Railton at 015 307 4893.

or

Human Research Ethics Committee
Prof. Cleaton-Jones
Administrator and Chair
University of the Witwatersrand
Wits Research Office
10th Floor Senate House
East Campus
Johannesburg
Tel: 011 717 1234
Fax: 011 717 1265

Do you understand what you have read/has been read to you? Please cross an answer below?

☐ Yes ☐ No

Do you agree to participate in this study? Please cross an answer below?

☐ Yes ☐ No

________________________ ________________________________

Participant Name Date

________________________ ________________________________

Participant Signature Date

Please obtain a signature from an adult witness if the participant is unable to read and write.

________________________ ________________________________

Witness Name Date

________________________ ________________________________

Witness Signature Date

________________________ ________________________________

Interviewer Name Interviewer Surname

________________________ Date _________-_________-20_________

Interviewer Signature

Do you understand what you have read/has been read to you? Please cross an answer below?

☐ Yes ☐ No

Do you agree to participate in this study? Please cross an answer below?

☐ Yes ☐ No

_________________________ _________________________________

Participant Name Date

_________________________ _________________________________

Participant Signature Date

Please obtain a signature from an adult witness if the participant is unable to read and write.

_________________________ _________________________________

Witness Name Date

_________________________ _________________________________

Witness Signature Date

_________________________ _________________________________

Interviewer Name Interviewer Surname

_________________________ Date _________-_________-20_________

Interviewer Signature

EVALUATION OF WARD-BASED OUTREACH TEAMS

Formal Signed Consent Form for Audio Recording

The researchers have my permission to audio record this interview/ focus group discussion (circle as appropriate) in order to include all important information. This has been explained to me in the participant information sheet. The evaluation has been explained to me and all my questions were answered to my satisfaction.

_________________________ _________________________________

Participant Name Date

_________________________ _________________________________

Participant Signature Date

Please obtain a signature from an adult witness if the participant is unable to read and write.

_________________________ _________________________________

Witness Name Date

_________________________ _________________________________

Witness Signature Date

_________________________ _________________________________

Interviewer Name Interviewer Surname

_________________________ Date _________-_________-20_________

Interviewer Signature

<u>EVALUATION OF WARD-BASED OUTREACH TEAMS</u>

Formal Signed Consent Form for Audio Recording

The researchers have my permission to audio record this interview/ focus group discussion (circle as appropriate) in order to include all important information. This has been explained to me in the participant information sheet. The evaluation has been explained to me and all my questions were answered to my satisfaction.

________________________ ________________________________

Participant Name Date

________________________ ________________________________

Participant Signature Date

Please obtain a signature from an adult witness if the participant is unable to read and write.

________________________ ________________________________

Witness Name Date

________________________ ________________________________

Witness Signature Date

________________________ ________________________________

Interviewer Name Interviewer Surname

________________________ Date _________-_________-20_________

Interviewer Signature

Interviewer Script: I will now ask you a few questions about your experience with community health workers

▪ **What are your experiences with the CHWs?**<br>▪ [probe]: Do CHWs visit the clinic?<br>▪ [probe]: Have you experienced any specific challenges with the CHWs?

▪ **How do the CHWs affect your workload?**<br>▪ [probe]: Do you find CHWs useful in the system?<br>▪ [probe]: What could they do for you?

Interviewer Script: I will now ask you a few questions about your roles and experiences as a social worker

**1. What are your roles in the community?**<br>▪ [probe]: How manageable do you find your job at present?<br>▪ [probe]: What are some of the challenges that you face?<br>▪ [probe]: What can be done to improve the challenges?

Interviewer Script: Thank you very much for your time and opinions.
